# Systematic review of nonlinear associations between the built environment and walking in older adults

**DOI:** 10.1186/s12889-025-25141-6

**Published:** 2025-12-11

**Authors:** Jie Shen, Junhang Fan, Yang Peng, Wenting Lu, Yajing Li, Xi Xu, Yuanbo Fei

**Affiliations:** 1 School of Urban Construction, Wuhan University of Science and Technology, Wuhan, China; 2 Hubei Provincial Engineering Research Center of Urban Regeneration, Wuhan University of Science and Technology, Wuhan, China; 3 Wuhan Natural Resources Conservation and Utilization Center, Wuhan, China; 4 Hubei General Institute of Planning and Design Co., LTD., Wuhan, China

**Keywords:** Built environment, Older adults, Walking activity, Influencing factors, Nonlinear effects, Threshold effects

## Abstract

**Background:**

In the context of global population aging, walking has become a key form of daily physical activity for older adults. Walking supports social participation and independence, and contributes significantly to both physical and mental health. However, there is still no broad consensus on which built environment factors influence older adults’ walking, or how these factors exert their effects.

**Methods:**

This study systematically searched five databases: MEDLINE, Web of Science, PubMed, Scopus, and Embase. Inclusion and exclusion criteria were defined using the PICOS framework. The review followed PRISMA guidelines, and studies were summarized according to their characteristics, methodological features, and built environment variables. The 46 included studies were assessed for quality using the National Institutes of Health’s Quality Assessment Tool for Observational Cohort and Cross-Sectional Studies. Based on this evaluation, a quantitative analysis identified 11 built environment factors with strong consensus regarding their influence on walking activity among older adults.

**Results:**

This study systematically reviewed 46 empirical studies to examine the impacts and mechanisms of the built environment on walking activity among older adults. The results indicate that the availability of retail stores, perceived safety, and aesthetic appeal significantly promote walking. In contrast, factors such as population density, residential density, land-use mix, intersection density, green view ratio, sidewalk density, distance to parks, green spaces, and plazas, as well as transit stop density, exhibit nonlinear effects. The nonlinear relationships involving sidewalk density and the distance to parks, green spaces, and plazas remain contested. Specifically, the following thresholds warrant attention: the optimal range of population density remains inconclusive and requires further validation; land-use mix is most effective within 0.55–0.7; intersection density is optimal at 25–65/km^2^; a streetscape green view index of about 24% yields the greatest effect; residential density of 10,000–25,000 households/km^2^ provides the strongest support for walking; sidewalk density of approximately 6 km/km^2^ is particularly relevant; the acceptable maximum walking distance to parks, green spaces, or plazas is 1–1.3 km; and transit stop density is preferable at 6–7 stops/km^2^. In addition, sufficient neighborhood shops and convenience facilities are needed to strengthen motivation for walking. Safety and aesthetic appeal play a key role in promoting walking by enhancing environmental perception and psychological sense of safety.

**Conclusions:**

Future research should adopt more longitudinal designs to overcome the limited causal inference of cross-sectional studies and to explore methods for controlling self-selection bias. In addition, future studies should strengthen the investigation of nonlinear relationships between the built environment and walking activity among older adults, with a focus on fine-grained analyses at the micro level. Integrating AI, big data, and machine learning for dynamic prediction and mechanism modeling will further help to uncover the complex interactions between the built environment and walking behavior. The findings of this study are consistent with the World Health Organization’s (WHO) core concept of “age-friendly cities” and provide actionable theoretical and practical guidance for human-centered, age-friendly urban design.

**Supplementary Information:**

The online version contains supplementary material available at 10.1186/s12889-025-25141-6.

## Introduction

Population aging has become an increasingly prominent global issue. According to United Nations data, by 2030 one in six people worldwide will be aged 60 years or older. Between 2020 and 2030, the global population aged 60 years and older will increase from 1 billion to 1.4 billion. By 2050, the number of people aged 60 and older is projected to double, reaching 2.1 billion. From 2020 to 2050, the population aged 80 years and above is expected to triple, reaching 426 million [1]. With advancing age, health problems among older adults become particularly pronounced. This trend not only increases the demand for healthcare services among older adults but also imposes severe limitations on their daily functioning, thereby undermining independence and quality of life.

Walking is the most common and safest form of physical activity among older adults. It not only fulfills daily mobility needs but also promotes social interaction and enhances physical vitality. Walking also serves as an effective means to improve physical function and overall health. This low-intensity activity significantly contributes to the prevention and management of chronic diseases [2] and represents a key strategy in promoting healthy aging. Therefore, optimizing the built environment to accommodate the natural decline in older adults’ walking capacity is an effective strategy to address population aging, support the development of age-friendly cities, and safeguard seniors’ health.

Scholarly discussions on urban travel modes have a long history. From early urban critics such as Jane Jacobs to later New Urbanism planners, scholars consistently advocated for walkable streets and communities to enhance the role of walking in transportation. In the 1990 s, transportation scholars such as Cervero [3, 4] first proposed the 3D model of the built environment, suggesting that density, diversity, and design affect not only travel frequency but also travel modes and route choices. Ewing and Cervero subsequently expanded the 3D model by incorporating destination accessibility and transit proximity, thereby developing the widely applied 5D model [5]. This framework has since become a major theoretical foundation for research on walking activity. During the same period, public health researchers found that the decline in physical activity, particularly walking, was linked to a range of health problems [6, 7]. They actively sought interdisciplinary collaboration to promote physical activity and improve population health.

Against this backdrop, Saelens introduced the concept of “walkability” [8], drawing on research in the built environment. The concept emphasizes enhancing community walkability to encourage walking and thereby improve physical health. He argued that certain environmental factors strongly influence residents’ willingness to walk. Following the introduction of walkability, numerous empirical studies emerged. Studies found that higher building density [9] and greater land-use mix [10] provide diverse destinations and shorten travel distances, both of which are positively associated with walking activity. Higher street network density [11] and intersection density [12, 13] not only increase route flexibility but also promote walking by improving visual coherence. These findings demonstrate that built environment factors substantially shape travel behavior and significantly influence walking across multiple dimensions, providing a foundation for subsequent research on the built environment and walking.

As research progressed, the socio-ecological model was gradually introduced into studies of the built environment and walking. This model emphasizes measuring external variables in real-world contexts to account for their effects on dependent variables [14]. This framework set new goals for empirical research: to test whether the influence of environmental factors on walking behavior remains significant after further controlling for socioeconomic factors such as education level and overall community economic conditions. Liang et al. [15] found that community environments exert not only direct effects on walking but also indirect effects on physical activity by enhancing individuals’ intentions to exercise. Even after accounting for education, health status, and psychological factors such as self-efficacy, environmental effects remained significant. Using large-scale travel survey data from Hong Kong, Yang et al. [16] applied geographically weighted regression to reveal spatial heterogeneity in the influence of the built environment on walking propensity. Factors such as population density, land-use mix, streetscape greenery, and transit accessibility exhibited varying effects across locations, yet their overall influence consistently outweighed that of individual socioeconomic variables. Together, these studies demonstrate that even after controlling for socioeconomic factors, built environment variables remain prominent determinants of walking behavior, showing consistent significance across diverse sociocultural and urban contexts. Therefore, this study focuses on the relationship between built environment factors and walking activity, grounded in their consistently central role compared with other socioeconomic variables.

Given the accelerating global aging process, some scholars have begun to examine how the built environment affects walking activity among older adults, a population with distinct needs. Studies have shown that population density is positively associated with walking activity in older adults, as denser areas provide greater opportunities for social interaction [17–19]. Areas with a high degree of land-use mix integrate diverse spatial functions, concentrating services, recreational facilities, and healthcare resources, thereby enhancing walking convenience for older adults [20, 21]. Higher intersection density improves street network connectivity, shortens walking distances, reduces physical exertion, and positively influences walking activity among older adults [22, 23]. Other scholars have found that some built environment factors exert varying effects on older adults’ walking activity. High population density can promote walking activity in older adults by enhancing social interaction [24, 25]. However, once density exceeds a certain threshold, resulting congestion and pollution reduce comfort and inhibit walking behavior [19]. Streetscape greenery, measured as the green view index, enhances visual comfort, alleviates fatigue during walking, and extends walking duration among older adults [16, 26]. However, once the green view index reaches a threshold, its positive effect plateaus or may even turn inhibitory. Collectively, these studies suggest that certain built environment factors may exhibit nonlinear relationships and threshold effects in influencing walking activity among older adults. However, existing research still lacks systematic synthesis of the potential nonlinear mechanisms and threshold effects of highly consensual built environment factors.

In summary, although many studies have examined the influence of built environment factors on walking activity among older adults, the existing literature still has several limitations. First, most existing studies on the built environment and walking focus on the general population, with limited in-depth analysis of older adults as a distinct group. Systematic reviews on this topic are also scarce. Second, although some studies have examined older adults specifically, findings on how built environment factors influence their walking remain inconsistent. Further clarification is needed to identify which factors show higher levels of consensus in affecting walking activity among this population. Third, the complex nonlinear mechanisms linking built environment factors to walking activity in older adults require further investigation.

Therefore, this review aims to systematically synthesize empirical studies, quantitatively analyze highly consensual built environment factors influencing older adults’ walking activity, examine their promoting and inhibiting effects, and explore potential nonlinear relationships. It further seeks to identify and quantify built environment factors that influence walking in nonlinear ways and to determine their optimal thresholds. This study not only deepens the understanding of the complex relationship between the built environment and walking behavior in older adults but also provides a solid theoretical foundation for “precision planning” and “age-friendly retrofitting.” By revealing the nonlinear relationships between built environment elements and walking activity, it identifies key intervention points and effective ranges, thereby preventing resource waste and enabling policymaking and urban design practice to shift from general principles to more fine-grained guidance.

The specific research questions are as follows: (1) Which factors demonstrate higher levels of consensus in studies on the built environment and walking activity among older adults? (2) Do these factors exert promoting, inhibiting, or nonlinear effects? (3) How do the effects of different built environment factors on older adults’ walking activity vary across thresholds?

## Methods

This review followed the PRISMA 2020 guidelines for systematic reviews [27] and was registered in the International Prospective Register of Systematic Reviews (PROSPERO) under registration number CRD420251088955.

### Search strategy

To systematically review studies examining the relationship between built environment features and walking activity among older adults, we searched five electronic databases: MEDLINE, Web of Science, PubMed, Scopus, and Embase. In addition, grey literature was retrieved from Google Scholar and ProQuest.

The search strategy combined subject headings and free-text terms, and was refined through multiple rounds of preliminary screening. Following the database searches, supplementary manual searches were conducted when necessary, including screening the reference lists of included studies to identify potentially relevant research that might have been missed. The search was conducted up to December 31, 2024. For example, the detailed PubMed search strategy is presented in Table 1. Search terms for the other databases are provided in Appendix 1.

**Table 1:** Pubmed search policy

Serial No.	Search contents
#1	built environment* OR urban environment OR environment design OR urban design OR neighborhood* OR pedestrian environment OR street OR physical environment OR walkability OR residential environment OR community environment
#2	walking activity OR physical activity OR mobility OR walking behavior
#3	older adult* OR elderly OR seniors OR aged OR older people OR older persons OR older citizens OR late life
#4	#1 AND #2 AND #3

### Study selection criteria

#### Population

Because definitions of older adults vary across studies—for example, ≥ 65 years in many developed Western countries and ≥ 60 years in most developing countries—this review uniformly applied ≥ 60 years as the minimum age criterion to ensure inclusion of studies from diverse contexts. Studies were included if the majority of participants were aged ≥ 60 years. Studies were excluded if they focused solely on younger populations or if the age distribution was unclear and could not be verified.

#### Intervention

This review focuses on built environment exposure factors that may influence walking activity among older adults. These factors include both objective and perceived neighborhood environments, such as street connectivity, land-use mix, population density, green space coverage, safety, and aesthetic appeal. Exposure was measured either objectively (e.g., GIS-based indicators, remote sensing data, or street audits) or subjectively (e.g., self-reported perceptions obtained through surveys or interviews). Studies were included if they examined at least one built environment characteristic in relation to walking activity among older adults.

#### Comparator

The comparison group was defined as exposure to different levels of built environment factors. For example, some studies compared older adults living in high-density versus low-density neighborhoods. However, the inclusion criteria of this review did not require studies to have an explicit comparison group; instead, the key criterion was whether the study analyzed the relationship between built environment factors and walking activity among older adults.

#### Outcome measures

The primary outcome of this review was walking activity among older adults, assessed in terms of walking frequency, duration, or intention. Outcome measures included both objective indicators (e.g., accelerometers, GPS tracking, GIS-based accessibility metrics) and subjective indicators (e.g., self-reported perceptions of walking activity). Studies were included if they reported at least one outcome directly related to walking activity.

#### Study design

This review primarily included empirical studies based on real-world data that explicitly reported sample characteristics, study design, variable measurement methods, and main findings. Studies that did not provide quantitative data—such as qualitative research, reviews, conference abstracts, preprints, and dissertations—were excluded. Only studies published in English were included.

### Data management and selection procedure

All retrieved records were first imported into Zotero for centralized management, then exported to Excel, where duplicate entries were identified and removed using the built-in deduplication function. The deduplicated records were then managed and screened in Excel. Based on the predefined inclusion and exclusion criteria, titles and abstracts of all records were reviewed to preliminarily exclude studies that did not meet the criteria. For studies that appeared potentially eligible, full texts were retrieved and screened again. Full-text screening was conducted according to the same criteria, and the final set of studies was determined for inclusion in the review. The entire screening process was conducted independently by the researchers, with repeated cross-checks of uncertain records to ensure accuracy and consistency.

Title and abstract screening was conducted by two primary reviewers (JS and JF) in collaboration with WL. Disagreements were resolved through discussion; if consensus could not be reached, a third author (YP) adjudicated. Full-text screening was performed independently by JS and JF. Throughout the screening process, the team met regularly to discuss uncertainties in applying the eligibility criteria. When a study’s eligibility remained disputed, YP and WL re-evaluated the article and issued the final decision.

Data extraction was performed independently by two primary reviewers (JS and JF) to ensure consistency and accuracy. Any discrepancies during extraction were resolved through discussion. The reviewers followed a transparent, systematic process, cross-checking each step and discussing issues as needed. Extracted information included authors and year of publication; study population; sample size; study design; definitions and measures of built environment and walking variables; analytic methods; and main findings. When inconsistencies remained, a third author (YP) verified the extracted data.

### Study quality assessment

The quality of each included study was assessed using the National Institutes of Health (NIH) Quality Assessment Tool for Observational Cohort and Cross-Sectional Studies. Each study was evaluated with a 14-item assessment. For each item, a “Yes” response was scored as 1, while “No,” “Not reported,” “Cannot determine,” or “Not applicable” were scored as 0. Total scores ranged from 0 to 14, with higher scores indicating higher study quality. Quality assessment results were used to gauge the reliability of included studies but were not applied as criteria for study inclusion. Detailed criteria and results of the quality assessment are provided in Appendix 2.

## Results

**Fig. 1:**
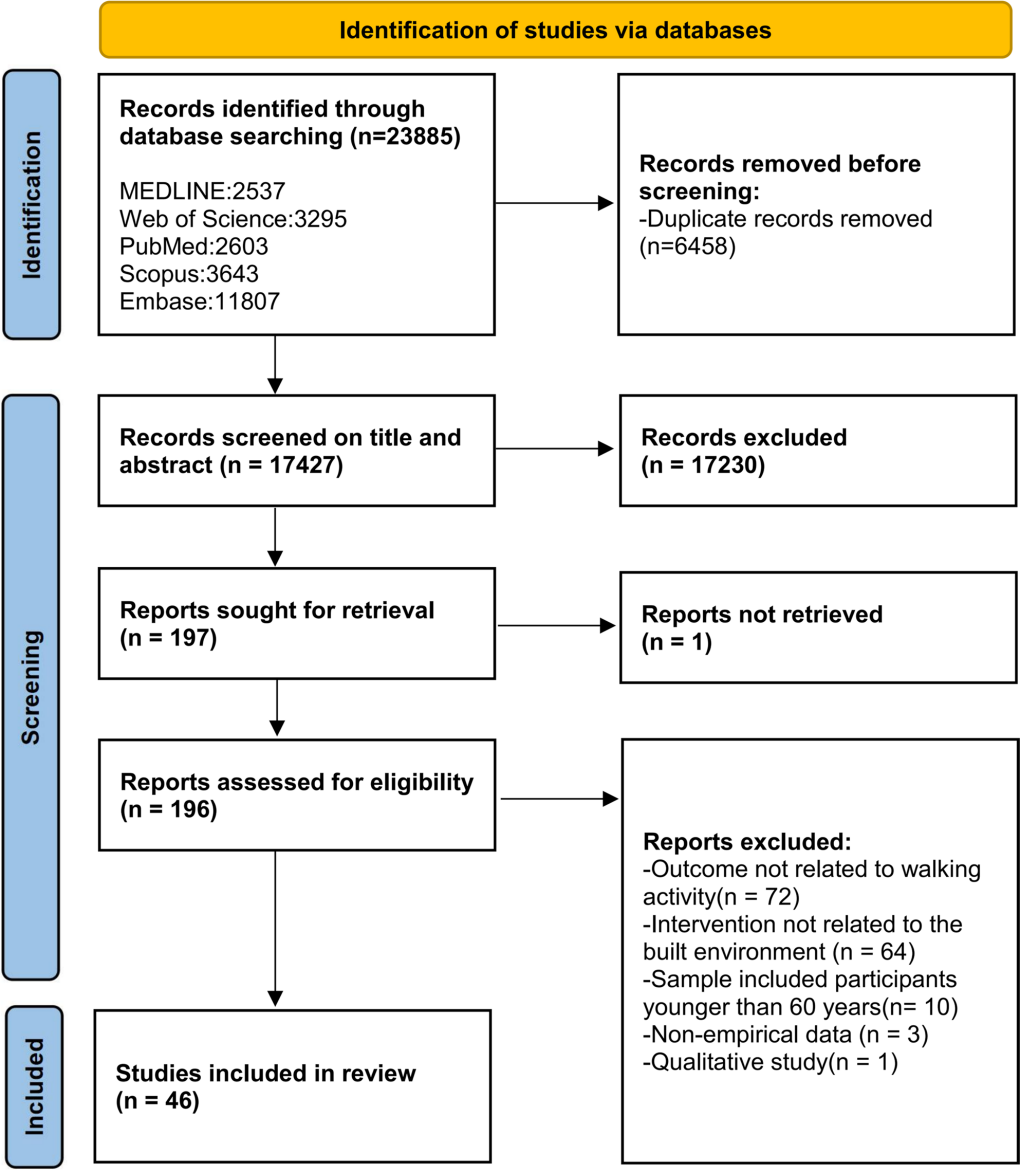
PRISMA 2020 Flow Diagram of the Study Selection Process

**Fig. 2:**
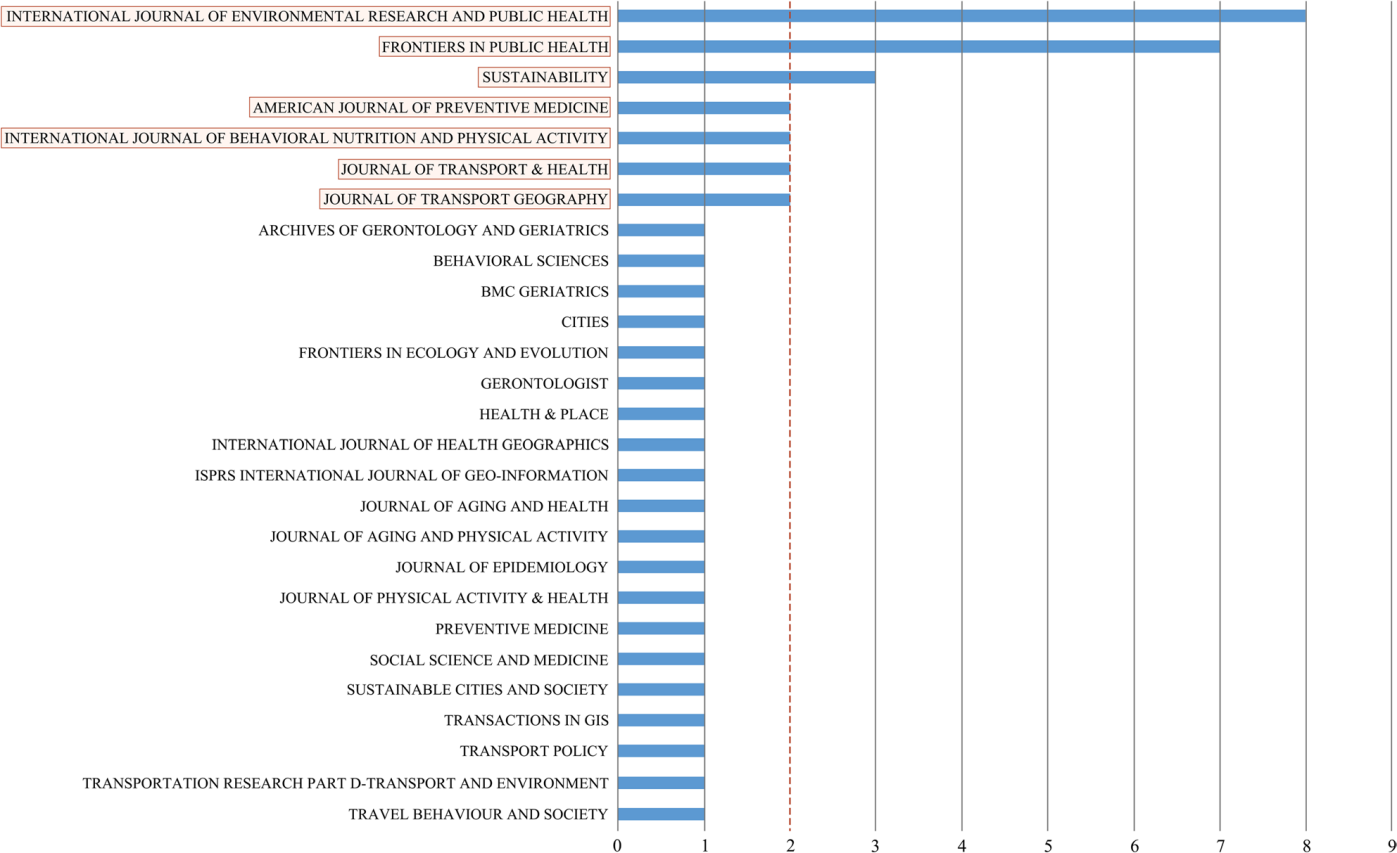
Distribution of Included Studies Across Academic Journals

Figure 1 presents the PRISMA flow diagram for this review. Table 2 summarizes the basic information and main findings of the included studies. The searches yielded a total of 23,897 articles (MEDLINE: *n* = 2,537; Web of Science: *n* = 3,295; PubMed: *n* = 2,603; Scopus: *n* = 3,643; Embase: *n* = 11,807; manual searches: *n* = 12). The included studies were published primarily in seven journals: *International Journal of Environmental Research and Public Health*, *Frontiers in Public Health*, *Sustainability*, *American Journal of Preventive Medicine*, *International Journal of Behavioral Nutrition and Physical Activity*, *Journal of Transport & Health*, *and Journal of Transport Geography* (Fig. 2).

**Table 2:** Summary of Included Studies

**NO.**	**Author**	**Year**	**Study Location**	**Spatial Scale**	**Sample Size**	**Built Environment Measurement Method**	**Walking Activity Measurement Method**	**Built Environment Variables**	**Walking Activity Variables**	**Analytical Model**	**Main Findings**
1	Dian Zhu, Dongjing Song [63]	2024	China	Community	169,996	1. Mobile signaling data from telecommunication companies 2. Geographic Information System (GIS) data 3. Baidu Street View images 4. Big data from real estate websites	Mobile signaling data	**Objective indicators:** property management fees, housing prices, year of construction, distance to the nearest park, land-use mix, number of sports facilities, street connectivity, and parking space ratio **Subjective perceptions:** sense of affluence, sense of boredom	Walking time	Random Forest (RF)	1. Distance to parks: Walking time increases when parks are located within 1,300 meters, but decreases significantly once this distance is exceeded.
2	Renjiang Xiong, Hang Zhao [83]	2024	China	Community	436	1. GIS data 2. Population data from the National Bureau of Statistics 3. Topographic data from the Ministry of Natural Resources	1. Survey questionnaire 2. Average daily walking distance estimated from Baidu Maps routing analysis	**Objective: **slope, population density, road network density, land-use mix, density of recreational facilities, distance to the nearest transit stop.	Walking distance	Mixed Geographically Weighted Regression (MGWR)	1. Slope shows a significant negative correlation with walking distance among older adults. 2. Population density, density of recreational facilities, and distance to the nearest transit stop are significantly positively associated with walking distance. 3. Land-use mix exerts a positive influence on walking distance. 4. When the road network density coefficient ranges from 0.02 to 0.24, walking distance increases most markedly; however, beyond 0.24, a declining trend emerges.
3	Congjian Chen, Yang Cao [42]	2024	China	Community	3,239	1. GIS data 2. Remote sensing image classification 3. Field survey	questionnaire survey	**Objective indicators:** land-use mix, residential density, length/density/connectivity of pedestrian streets, accessibility of public service facilities, accessibility of green spaces, accessibility of public transport	walking frequency	Hierarchical Linear Model (HLM)	1. Land-use mix, street connectivity, and accessibility to green spaces/public service facilities significantly promote walking activity and improve BMI. 2. Excessively high residential density suppresses walking.
4	Jiani Wu, Chaoyang Li [59]	2024	China	Community	4,329	Zhongshan Household Travel Survey (2012) + Land-use data from the Urban Planning Bureau	Travel survey questionnaire	**Objective indicators:** population density, land-use mix, transit stop density, commercial accessibility, sidewalk density, accessibility of green spaces	walking time	Gradient Boosting Decision Trees (GBDT)	1. Walking increases significantly when population density is ≥30,000 persons/km² or land-use mix is ≥0.6. 2. For younger older adults, the threshold of transit stop density that promotes walking is 7.8 stops/km², whereas for older-old adults it is 6 stops/km². 3 Green space coverage: walking time increases significantly when coverage ranges from 10% to 47%, but the effect diminishes once it exceeds 47%. 4. Sidewalk density has a negative effect on walking duration when between 0–6 km/km², but turns positive beyond this threshold.
5*	Jonathan R. Olsen, Elise Whitley [66]	2024	the UK	Community	6,450	1.GIS data 2.Ordnance Survey data	IPAQ-SF	**Objective:** street connectivity, residential density, accessibility of green spaces, recreational facilities, public transport, number of retail facilities, crime rate**Subjective:** perceived safety	walking time	Multilevel Least Squares Regression Model	Longitudinal study (combining Waves 2 and 5 of prior physical activity and walking data): 1. Previous exercise habits and leisure walking significantly predicted walking levels several years later. 2. Having more close friends and greater availability of retail facilities promoted walking. 3. Areas with higher crime rates were paradoxically associated with more walking, possibly due to characteristics of high-density urban areas
6	Peng Zang, Hualong Qiu [84]	2023	China	Community	597	1. ArcGIS data 2. Baidu Street View images 3. Remote sensing imagery	IPAQ-LC (International Physical Activity Questionnaire–Long Form)	**Objective indicators: **Green View Index (GVI), Normalized Difference Vegetation Index (NDVI), population density, land-use mix, street connectivity, intersection density, number of transit stops and distance to the nearest stop	Walking propensity	Random Forest (RF)	1.Green View Index (GVI) was identified as the most important variable (15.7%), with an optimal threshold of approximately 0.24. 2. The NDVI threshold was around 0.45, beyond which walking was suppressed. 3. Land-use mix had a positive effect when below 0.45, but an excessive level exerted a negative effect. 4. High street connectivity and overly dense intersection density were found to inhibit walking. 5. The number of transit stops and moderate distances exerted positive effects on walking.
7	Yang Cao, Hao Wu [43]	2023	China	Block	1,275	1. GIS/POI data 2. Questionnaire survey 3. Remote sensing imagery	Questionnaire survey	**Objective indicators: **road network accessibility, intersection density, density of public service facilities, density of green spaces, density of commercial facilities **Subjective indicators:** perceived road safety, adequacy of night-time lighting, comfort of natural landscapes, comfort of cultural landscapes	Participation in leisure walking (yes/no), Meeting the threshold of ≥150 minutes/week (yes/no)	Hierarchical Linear Model (HLM)	1. The core range for leisure walking among older adults is within an 800 m radius, followed by the outer ring of 800–1500 m. 2. Park density and accessibility significantly promote walking, particularly within the outer ring (800–1500 m). 3. High residential density, uneven road surfaces, and low comfort of natural landscapes significantly suppress walking. 4. Night-time lighting and road safety exert positive effects on walking.
8	Erica Twardzik, Jason R. Falvey [85]	2023	United States	Block	4,836	NaNDA database	NHATS survey questionnaire	**Objective indicator: **public transit stop density	whether the respondent engaged in walking exercise during the past month	Logistic Regression Model	1. Older adults residing in areas with more than 10 transit stops per square mile exhibited significantly higher walking intention. 2. Social network size, educational attainment, and race/ethnicity (Hispanic and other minority groups) significantly promoted walking, whereas mobility impairments and low social cohesion suppressed walking.
9	Qinglin Jia, Tao Zhang [67]	2022	China	Community	863	1. ArcGIS data 2.Baidu Map POI	Questionnaire	**Objective indicators: **residential density, land-use mix, number of pedestrian crossing facilities, number of transit stops, number of retail facilities, number of food and beverage establishments, recreational facilities, public service facilities	walking frequency	Multiscale Geographically Weighted Regression (MGWR)	1. The MGWR model achieved the best fit (Adj. R² = 0.949), outperforming both OLS and GWR models. 2. Retail and food facilities promoted walking; however, excessive clustering or high population density may suppress walking due to crowding. 3. The effects of transit stops exhibited marked regional variation.
10	Yongjiang Yang, Kuniaki Sasaki [86]	2022	Japan	Community	2,003	1. ArcGIS data 2. National Land Information Database	Travel diary (PTS survey)	**Objective indicators: **population density, road density, number of transit stops, distance to the nearest railway station, number of medical facilities, distance to sports facilities, number of parks, land-use mix	walking frequency	Ordered Logistic Regression Model	1. Population density and land-use mix were positively associated with walking frequency. 2. Distance to railway stations had a significant negative effect on both walking frequency and duration. 3. The number of transit stops exerted a negative effect on both walking frequency and duration. 4. The number of medical facilities promoted walking.
11	Chunmei Yang, Xianglong Tang [87]	2022	China	Community	10,700	1. ArcGIS data 2.Google Street View (GSV)	Hong Kong Travel Characteristics Survey (HKTCS)	**Objective indicators: **population density, land-use mix, street intersection density, streetscape greenery (green view index), accessibility of transit stops	Walking propensity	Logistic Regression Model & Geographically Weighted Logistic Regression (GWLR)	1.**Global results:** population density, land-use mix, streetscape greenery (green view index), and accessibility of transit stops all showed significant positive effects, while intersection density had no effect.
12	Mohammad Paydar, Asal Kamani Fard [38]	2022	Chile	Community	463	1. ArcGIS data 2. Audit (revised versions of PEDS and SPACES)	EOD (Every Other Day) travel diary	**Objective indicators: **population density, housing density, land-use mix, destination accessibility (commercial, educational, medical, parks, etc.), street connectivity, slope, sidewalk characteristics (width, quality, obstacles), greenery, building height, façade details, landscape visibility **Subjective indicators: **lighting, perceived safety, perceived aesthetics	walking time	Hierarchical Multiple Regression Model	1.**Positive associations:** land-use mix, sidewalk length, number of parks/squares, natural landscape visibility, building height, and building façade details. 2.**Negative associations:** number of educational facilities, street connectivity, and number of surface parking lots.
13	Linchuan Yang, Xianglong Tang [88]	2022	China	Community	11,732	1.ArcGIS data 2.OSM	TSXR travel diary	**Objective indicators:** population density, land-use mix, intersection density, distance to commercial centers, transit route density	walking time	Seemingly Unrelated Regression Equations Model (SURE)	1. Land-use mix, intersection density, and transit route density had significant positive effects on both walking frequency and duration. 2. Distance to commercial centers exerted a significant negative effect on both indicators. 3. Population density showed no significant effect, suggesting a possible nonlinear relationship.
14	Mohammad Paydar, Javier Arangua Calzado [89]	2022	Chile	Community	1721	1.GIS 2. Municipal cadastral data	EOD (Every Other Day) travel diary (2013)	**Objective indicators: **residential density, population density, land-use mix, number of destinations, street connectivity, intersection density, street density, number of parks and squares, number of street trees, slope	walking time	Hierarchical Multiple Regression Model	1.**Overall walking: **positively associated with housing density, parks/squares, and land-use mix; negatively with educational facilities and street connectivity. 2.**Work-related walking: **promoted by residential density, parks/squares, and land-use mix; suppressed by traffic accidents, crime, and educational facilities. 3.**Education-related walking: **increased with more educational facilities, absence of a driver’s license, and higher education levels. 4.**Shopping-related walking:** facilitated by land-use mix and parks/squares; reduced by slope and Internet access; more prevalent among low-income and home-based workers.
15	Linchuan Yang, Yibin Ao [47]	2021	China	Community	10,700	1.Google Street View (GSV) 2.ArcGIS 3.OSM	2011 Hong Kong Travel Characteristics Survey (TCS)	**Objective indicators: **streetscape greenery (GVI), population density, land-use mix, intersection density, number of transit stops, number of recreational facilities	Walking propensity	Random Forest (RF)	1.**Streetscape greenery (GVI):** positive effect when GVI < 0.24; effect weakened or turned slightly negative beyond 0.24. 2.**Population density:** positive below 75,000 persons/km²; negative effects observed above this threshold. 3.**Land-use mix:** positive when < 0.55; negative within the 0.55–0.80 range. 4.**Intersection density:** promoted walking within 25–65/km²; excessively high or low levels were unfavorable. 5. Accessibility of transit stops and recreational facilities: also exhibited threshold effects.
16	Peng Zang, Hualong Qiu [90]	2021	China	Community	597	1.ArcGIS 2.sDNA	IPAQ-LC questionnaire	**Objective indicators: **land-use mix, street connectivity, intersection density, NDVI, POI density, number and distance of bus/metro stations, number of pedestrian overpasses, road intersection density	walking time	Stepwise Regression Model	1.**Low-density areas:** transport-related walking was positively associated with land-use mix, transit stop density, and NDVI. 2.**Medium-density areas:** leisure walking was positively associated with educational POI density, intersection density, and NDVI. 3.**High-density areas:** leisure walking was negatively associated with NDVI, street connectivity, and intersection density.
17	Linchuan Yang, Jixiang Liu [91]	2021	China	Community	1083	1.Google Street View (GSV) 2.ArcGIS 3.OSM	2011 Hong Kong TCS supplementary survey	**Objective indicators: **streetscape greenery (GVI at 400 m, 800 m, and 1600 m), population density, land-use mix, road intersection density, number of metro stations, number of recreational facilities	walking time	Linear Regression Model& Box–Cox Transformed Model& Geographically Weighted Regression (GWR)	1. Streetscape greenery (GVI) at all three buffer scales (400 m, 800 m, 1600 m) had a consistently significant positive effect on walking duration. 2. Accessibility of recreational facilities showed a stable and positive association with walking duration.
18	Jiani Wu, Chunli Zhao [30]	2021	China	Community	4,784	GIS	2012 Zhongshan Household Travel Survey (ZHTS)	**Objective indicators: **population density, land-use mix, sidewalk density, transit stop density, commercial accessibility, proportion of green space	walking frequency	Generalized Additive Mixed Model (GAMM)	1.**Threshold effects:** population density had a positive effect below 7,000 persons/km², but the effect diminished or disappeared beyond this level; land-use mix showed a threshold of 0.65, above which the effect turned negative; sidewalk density significantly promoted walking once it exceeded 6 km/km²; transit stop density showed a saturation effect at around 6 stops/km²; green space ratio promoted walking within the 8–40% range but exerted negative effects above 40%. 2.**Commercial accessibility:** exhibited a largely linear positive effect.
19	Takafumi Abe, Kenta Okuyama [92]	2021	Japan	Community	590	GIS	Healthwalk, Kao Corp	**Objective indicators:** Hilliness, mean land slope	Walking speed	Multiple Linear Regression Model	1.Slope was significantly negatively associated with walking speed.
20	Long Cheng, Kunbo Shi [58]	2021	China	Community	702	GIS	2013 Nanjing Household Travel Survey	**Objective indicators:** population density, land-use mix (residential, commercial, educational, recreational, and public service uses), street connectivity (sidewalk length per built-up area), number of transit stops, number of bike-sharing stations, distance to the nearest park/square, distance to the nearest chess/poker room	walking time	Geographically Weighted Regression (GWR)	1. Population density and land-use mix were positively associated with walking in some areas, but showed negative effects in high-density CBD zones and in western districts with excessive land-use mixing. 2. Street connectivity, number of transit stops, and number of bike-sharing stations were significantly and positively associated with walking across all areas. 3. Distance to parks/squares was negatively associated with walking time. 4. Distance to chess/poker rooms was positively associated with walking time.
21	Lilah M. Besser, Diana P. Mitsova [93]	2021	United States	Block	72,753	1.NLCD 2011 2.ArcGIS	2017 National Household Travel Survey (NHTS) travel diary	**Objective indicators: **proportion of open space, proportion of forest cover, population density	walking time	Linear Regression Model	1. Higher proportions of forest cover were associated with increased walking time. 2. The proportion of open space showed no overall significant effect, but was positively associated with walking time among African American populations. 3. Among Hispanic populations, forest cover was negatively associated with walking time.
22	Peng Zang, Xuhong Liu [94]	2020	China	Block	180	1. Baidu Street View (BSV) 2.GIS (ArcGIS)	IPAQ-Long Form (IPAQ-LF)	**Objective indicators: **streetscape greenery (GVI), land-use mix, street connectivity, population density	walking time	Bivariate Correlation Analysis Model	1. GVI was positively associated with walking duration.
23	Florian Herbolsheimer, Atiya Mahmood [95]	2020	United States	Community	434	SWEAT-R (Senior Walking Environmental Audit Tool-Revised)	Telephone survey	**Objective indicators:** population density, building type (single-family vs. multi-family), street sidewalks (continuity, pavement quality), traffic safety features (zebra crossings, speed-reducing facilities), availability of parks/outdoor fitness facilities	walking time	Multilevel Logistic Regression Model	1. Older adults in high-density neighborhoods engaged more frequently in transport-related walking. 2. Safe street-crossing facilities, the presence of parks/fitness facilities, paved sidewalks, and diversity in building types all promoted transport-related walking.
24	Hui He, Tingting Li [96]	2020	China	Community	1,161	1. GIS (2010 Sixth National Population Census) 2. Wuhan Master Urban Plan 3. Baidu Maps 4. Gaode (Amap) Maps	Questionnaire (adapted from IPAQ)	**Objective indicators:** population density, street connectivity, land-use mix, retail store density	walking time	Multilevel Logistic Regression Model	1. Street connectivity was significantly positively associated with leisure walking.
25	Zachary J. Christman, Maureen Wilson-Genderson [97]	2020	United States	Community	2,224	Google Street View	Telephone survey	**Objective indicators: **sidewalk characteristics, land-use mix **Perceptual indicator: **aesthetics	walking time	Multilevel Linear Regression Model	1. Sidewalk quality was positively associated with both leisure walking and transport-related walking. 2. Land-use mix, commercial facilities, and parking availability were positively associated with transport-related walking, whereas single-family housing was negatively associated. 3. Neighborhood aesthetics were positively associated with leisure walking.
26	Long Cheng, Jonas De Vos [29]	2020	China	Community	702	GIS	Travel diary	**Objective indicators: **population density, land-use mix, street connectivity, number of transit stops, number of bike-sharing stations, distance to the nearest park/square, distance to the nearest chess/poker room	walking time	Random Forest (RF)	1. Population density promoted walking within the range of 6–20 persons per 1,000 m², but declined when exceeding 20 persons per 1,000 m². 2. A land-use mix between 0.4 and 0.7 was most favorable for walking, whereas higher levels exerted negative effects. 3. Street connectivity promoted walking when below 6 km/km², but became unfavorable at higher levels. 4. At least three transit stops were required to significantly promote walking; bike-sharing stations had positive effects within the range of 0–10, but showed no effect beyond 10. 5. Proximity to parks or squares within 1 km significantly promoted walking. 6. Shorter distances to chess/poker rooms were associated with reduced walking time.
27	Ester Cerin, Anthony Barnett [25]	2020	China	Community	909	GIS	NWQ-CS（Neighbourhood Walking Questionnaire for Chinese Seniors）	**Objective indicators:** residential density, street intersection density, food retail density, entertainment density, recreational facility density, public transport density, park area	walking frequency	Generalized Additive Mixed Models (GAMMs)	1. Residential density promoted transport walking. 2. Public transport density increased walking outside the community but reduced it inside. 3. Recreational facility density promoted leisure walking at medium levels (~12,000 households/km²), but declined when higher. 4. Street intersection density enhanced external walking but reduced internal leisure walking under high density. 5. Medium-to-high residential density (10,000–25,000 households/km²) was optimal; extremely high density had negative effects.
28	Tanja Brüchert, Pia Hasselder [98]	2020	Germany	Community	2,189	NEWS (Neighborhood Environment Walkability Scale, German version)	IPAQ-LF	**Objective indicators: **residential density, destination proximity (e.g., shops, pharmacies, banks, cafés), number of destinations accessible within a 20-minute walk, transit accessibility, pedestrian/cycling/sharing infrastructure, street connectivity **Perceptual indicators:** aesthetics, traffic safety	walking frequency	Logistic Regression Model	1. Almost all built environment (BE) factors were significantly positively associated with both walking participation and frequent walking. 2. Destination proximity showed the strongest effect, followed by transit accessibility. 3. Weekly walking duration was significantly positively associated only with residential density.
29	Zhengying Liu, Astrid Kemperman [71]	2020	China	Community	316	ArcGIS	7-day recall questionnaire	**Objective indicators:** Accessibility to shops (0–5, 6–10, 11–15, >15 minutes), Distance to the nearest park (<800 m, 800–1200 m, 1200–1600 m, >1600 m) **Perceptual indicators:** Satisfaction with neighborhood aesthetics, Satisfaction with walking path conditions, Satisfaction with traffic safety, Satisfaction with crime safety	walking time	Random Effects Ordered Logit Model (REOLM)	1. Walking duration was longer when the nearest park was located within 800 m, whereas distances of 1200–1600 m were significantly negatively associated with walking duration. 2. Higher satisfaction with aesthetics, walking path conditions, traffic safety, and crime safety was positively associated with walking duration.
30	Razieh Zandieh, Javier Martinez [73]	2019	the UK	Community	173	GIS	1. GPS data records 2. Questionnaire	**Objective indicators:** distance to green space, attractiveness of green space, size of green space, number of green spaces	walking time	Multilevel Linear Regression Model	The only factor significantly associated with walking was green space area, with larger areas linked to longer walking duration.
31	Camille Perchoux, Ruben Brondeel [40]	2019	Luxembourg	City	342	1.GIS 2. Remote sensing imagery 3. Luxembourg national database	VERITAS survey	**Objective indicators: **number of facilities, diversity of facilities, street connectivity, number of public transport stops, greenness index	Walking propensity	Multilevel Linear Regression Model	1. Street connectivity was positively associated with walking intention when below eight intersections, but turned negative beyond this threshold. 2. The number of public transport stops was negatively associated with walking intention as stop density increased. 3. Walking intention decreased significantly with increasing distance.
32	Tiina E. Laatikainen, Mohammad Haybatollahi [56]	2019	Finland	Community	844	1.PPGIS 2.GIS	1. PPGIS (Public Participation GIS) mapping 2. Self-reported travel mode and frequency	**Objective indicators: **distance to green space, attractiveness of green space, size of green space, number of green spaces	Walking distance	Ordinary Least Squares (OLS) Regression	1. Sidewalk density, residential density, intersection density, transit stop density, and sports facility density were all significantly and positively associated with walking distance.
33	Yiyang Yang, Dongsheng He [68]	2019	China	Block	10,700	1. Google Street View (GSV) imagery 2.PSPNet	Hong Kong Travel Characteristics Survey (HKTCS 2011–2012)	**Objective indicators: **streetscape greenery (GVI), population density, intersection density, land-use mix, number of retail stores, number of entertainment facilities, distance to the nearest metro station	walking time	Multilevel Logistic Regression Model & Multiple Linear Regression Model	1. Streetscape greenery (GVI) was significantly positively associated with both walking intention and walking duration. 2. Population density, number of retail stores, and proximity to the nearest metro station were positively associated with walking intention, whereas intersection density showed a negative association. 3. The number of entertainment facilities was positively associated with walking duration.
34	Razieh Zandieh, Johannes Flacke [99]	2017	the UK	Community	173	1.GIS 2.OS MasterMap 3.POI data	GPS tracking (i-gotU GT-600)	**Objective indicators:** residential density, land-use mix, land-use intensity (proportion of specific land types such as green space or industrial land), street connectivity, retail density	walking time	Hierarchical Linear Regression Analyses	1. The intensity of green space and recreational center use was positively associated with outdoor walking duration. 2. The intensity of industrial land use and school land use was negatively associated with walking duration. 3. Street connectivity was negatively associated with walking duration.
35	Yung Liao, Pin-Hsuan Huang [100]	2017	China	Community	1,032	IPAQ-E (International Physical Activity Questionnaire – Environmental Module, Chinese version)	IPAQ-LV (International Physical Activity Questionnaire – Long Version, Chinese telephone-based version)	**Objective indicators: **residential density, shop accessibility, public transport accessibility, presence of sidewalks, accessibility of recreational facilities, street connectivity, presence of destinations **Perceptual indicators: **perceived safety from crime at night, traffic safety, neighborhood aesthetics	Walking propensity	Binary Logistic Regression	1. The presence of sidewalks and destinations was significantly positively associated with both leisure and transport walking. 2. Shop accessibility and accessibility of recreational facilities were significantly positively associated with leisure walking. 3. Poor traffic safety was significantly negatively associated with transport walking.
36	Maruí W. Corseuil Giehl [74]	2016	Brazil	Community	1,637	1. NEWS-Brazil (modified version of the Neighborhood Environment Walkability Scale) 2. Social Support Scale	IPAQ – Long Form	**Perceptual indicators: **sidewalks, green spaces, street slope, litter, open drains, traffic barriers, zebra crossings, air pollution, street lighting, crime safety, social support, parks, walking trails, dog ownership and walking	walking time	Multinomial Logistic Regression Models	1. Good sidewalk conditions, the presence of zebra crossings, perceived daytime safety, street lighting, availability of parks/recreational facilities, and dog walking were all positively associated with transport walking. 2. Good sidewalk conditions, perceived daytime safety, and social support from family and friends were positively associated with leisure walking. 3. Litter pollution exerted a negative effect on transport walking.
37	Razieh Zandieh, Javier Martinez [101]	2016	the UK	Community	173	NEWS (modified version) questionnaire	GPS tracking (i-gotU GT-600)	**Objective indicator: **walking infrastructure **Perceptual indicators: **safety, aesthetics	walking time	Hierarchical Linear Regression Models	Perceived neighborhood safety, quietness, and aesthetics were all significantly positively associated with walking duration.
38	Jordana L. Maisel [102]	2016	United States	Community	121	NEWS（Neighborhood Environment Walkability Scale）	IPAQ（International Physical Activity Questionnaire）	**Objective indicators:** residential density, street connectivity, land-use mix**Perceptual indicators: **traffic safety, crime safety, aesthetics, neighborhood satisfaction	walking time	Logistic Regression Model	1. Street connectivity was significantly positively associated with overall walking time. 2. Aesthetics was positively associated with leisure walking.
39	Marui Weber Corseuil Giehl [103]	2016	Brazil	Community	1705	GIS	IPAQ – Long Form	**Objective indicators:** Population density, land-use mix, street density, street connectivity, paved streets, sidewalks, streetlights, area income, public open spaces	walking frequency	Multilevel Logistic Regression Model	1. Higher population density, street connectivity , sidewalk proportion , and paved streets significantly increased the likelihood of walking for transportation. 2. Walking for leisure was significantly associated only with higher area income and street density. 3. Built environment effects were domain-specific; transport-related walking benefited more directly from connectivity and infrastructure quality.
40	Yi Zhang, YuanLi, Qixing Liu [57]	2014	China	Community	4,308	1.GIS 2. Zhongshan Planning Bureau data	Self-reported travel survey (Zhongshan Household Travel Survey, 200)	**Objective indicators: **population density, land-use mix, sidewalk density, bus stop density, commercial accessibility, proportion of green space	walking time	Zero-Inflated Poisson (ZIP) Regression	1. Sidewalk density, bus stop density, commercial accessibility, and the proportion of green space were all significantly positively associated with walking frequency and duration. 2. Population density and land-use mix were negatively associated with walking frequency and duration.
41	Ester Cerin, Ka-yiu Lee [39]	2013	China	Block	484	EAST-HK (Environmental Assessment Tool – Hong Kong)	1. IPAQ-LC (Chinese version) 2. NWQ-CS (Chinese version)	**Objective indicators: **destination diversity, destination prevalence, walking infrastructure **Perceptual indicator:**street safety	walking time	Generalized linear models (GLMs)	1. Bus stop prevalence and recreational destination diversity were positively associated with walking time. 2. Perceived safety was negatively associated with walking time.
42	Kenji Tsunoda, Taishi Tsuji [72]	2012	Japan	Community	421	IPAQ-E (Japanese version)	PASE (Japanese version, Physical Activity Scale for the Elderly)	**Objective indicators: **residential density, accessibility of recreational facilities, presence of sidewalks, accessibility of public transportation, slope **Perceptual indicators: **traffic safety, crime safety, aesthetics	walking time	Logistic Regression Model	1. Traffic safety and aesthetics were significantly positively associated with walking time. 2. Accessibility of public transportation was significantly negatively associated with walking time.
43	Andrea Nathan, Gavin Pereira [104]	2012	Australia	Block	2,918	1.GIS 2.Sensis Yellow Pages	Active Australia Survey (self-reported)	**Objective indicators: **food retail, general retail, medical services, financial services, general services (e.g., pharmacies, hairdressers), social infrastructure (e.g., cafés, restaurants, places of worship), diversity of commercial destinations, street connectivity	walking time	Logistic Regression Model	1. General services (e.g., pharmacies, hairdressers) within 400 m and 800 m significantly increased the likelihood of weekly walking. 2. Social infrastructure (e.g., restaurants, churches) within 800 m was positively associated with weekly walking time.
44	Shigeru Inoue, Yumiko Ohya [105]	2011	Japan	Community	1,921	IPAQ-E (Japanese version)	Self-reported questionnaire	**Objective indicators: **residential density, shop accessibility, public transportation, bicycle lanes, sidewalks, accessibility of sports facilities **Perceptual indicators: **traffic safety, crime safety, social environment, aesthetics	walking time	Multilevel Logistic Regression Model	1. Social environment, aesthetics, bicycle lanes, accessibility of sports facilities, and the number of motor vehicles were significantly associated with transport walking. 2. Social environment and aesthetics were significantly associated with leisure walking.
45	Zhe Wang, Chanam Lee [106]	2010	United States	Community	114	1. Perception-based questionnaire 2.GIS	Retrospective questionnaire	**Objective indicators:** daylighting, window views, plot greenery, corner plots, plot walkability, number of neighborhood destinations, route choice, sidewalks, lighting, benches **Perceptual indicators:** crime safety, traffic safety	walking time	Multivariate Logistic Regression Models	1. Crime safety, the number of neighborhood destinations, availability of sidewalks, and adequate lighting were all significantly positively associated with walking frequency and duration. 2. Proximity to pharmacies was positively associated with longer walking duration, whereas proximity to medical facilities was negatively associated with walking duration.
46	Luis F. Gómez, Diana C. Parra [107]	2010	Colombia	Community	1,966	GIS	Modified IPAQ-Short Form	**Objective indicators: **park density, street connectivity, Ciclovía (bike lane network), number of TransMilenio stations, slope **Perceptual indicators: **perceived traffic safety, satisfaction with sidewalk quality	walking time	Multilevel Adjusted Models	1. Park density, perceived traffic safety, and the presence of Ciclovía corridors were significantly positively associated with walking time. 2. Street connectivity and slope were significantly negatively associated with walking time.

* indicates longitudinal study

**Fig. 3:**
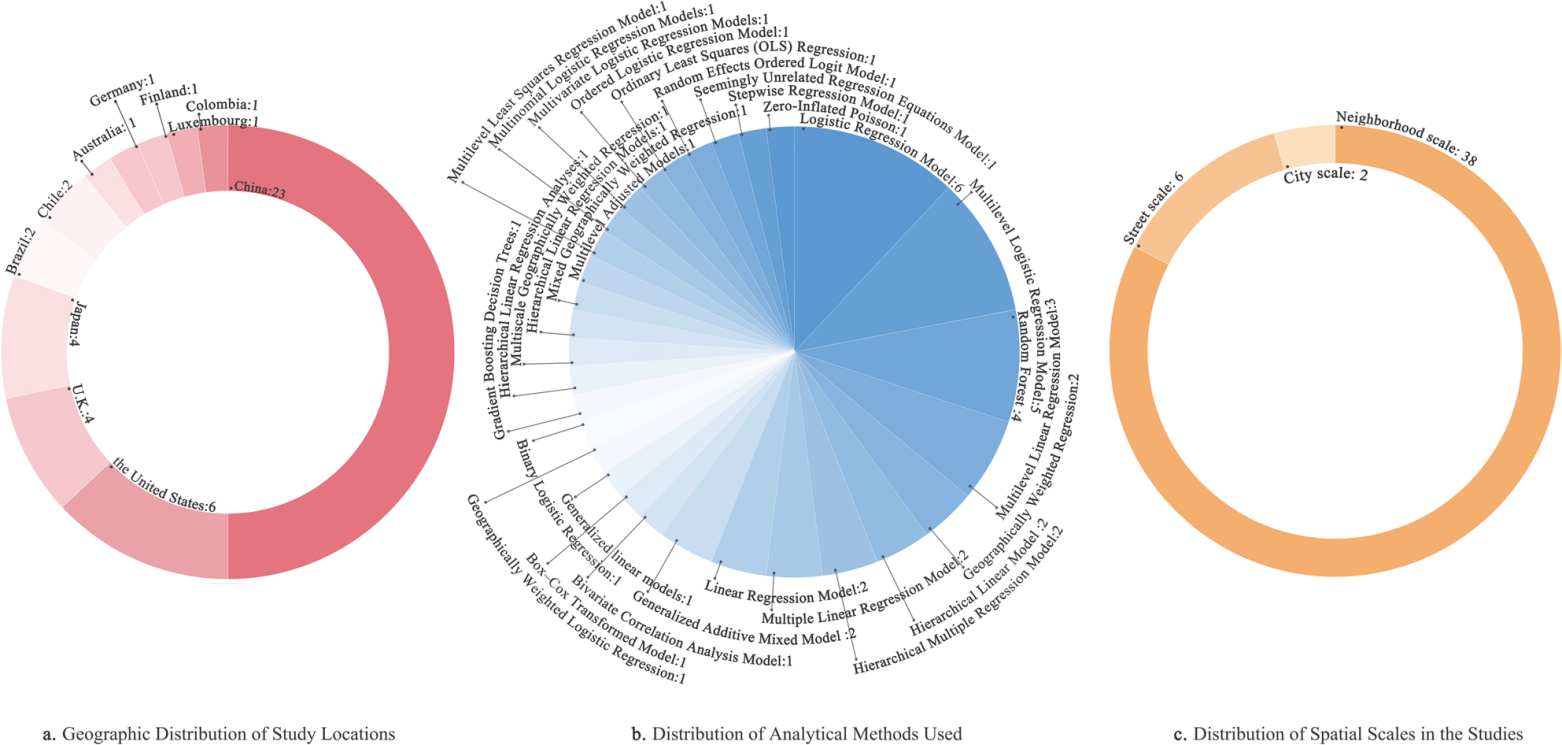
Summary of Research Characteristics in the Reviewed Literature. **a** Geographic distribution of study locations. **b** Distribution of analytical methods used. **c** Distribution of spatial scales in the studies

In terms of study location (Fig. 3a), most were conducted in China (*n* = 23), followed by the United States (*n* = 6), Japan (*n* = 4), the United Kingdom (*n* = 4), Brazil (*n* = 2), Chile (*n* = 2), Australia (*n* = 1), Germany (*n* = 1), Finland (*n* = 1), Colombia (*n* = 1), and Luxembourg (*n* = 1). Sample sizes of older adults ranged from 114 to 169,996.

In terms of methodology (Fig. 3b), current approaches to studying walking behavior have expanded from traditional regression models, which primarily test hypotheses, to machine learning and geospatial statistical methods that emphasize both predictive performance and mechanism exploration. This shift highlights the ongoing methodological advancement and interdisciplinary integration in research on walking among older adults. The first category consisted of regression models (*n* = 33), including logistic regression (e.g., logistic regression, multilevel logistic regression, ordered/multinomial), linear and multiple regression (e.g., multiple linear regression, OLS), hierarchical models (e.g., hierarchical linear/multiple regression, multilevel linear regression), and other forms such as SURE, stepwise regression, and zero-inflated Poisson models. These methods remain the dominant tools in built environment and walking research due to their strong interpretability and applicability. The second category was spatial econometric models (*n* = 5), primarily including geographically weighted regression and its extensions (GWR, MGWR, multiscale GWR, GWLR), which reveal the spatial heterogeneity of environmental influences on walking behavior. The third category comprised generalized semi-parametric models (*n* = 4), such as generalized linear models (GLM), generalized additive mixed models (GAMM), and Box–Cox transformations. These approaches introduce flexible functional forms or distributional assumptions within the regression framework to capture nonlinear relationships and complex effects. The fourth category was machine learning and related analytical methods (*n* = 5), including random forests (RF) and gradient boosting decision trees (GBDT), which can identify complex nonlinear patterns among variables in large datasets.

In recent years, research methods for addressing nonlinear relationships between the built environment and walking activity have expanded considerably. Models such as generalized additive mixed models (GAMM), gradient boosting decision trees (GBDT), and random forests (RF) have been widely applied to identify complex nonlinear effects. Random forests (RF) [28], as an ensemble machine learning algorithm combining multiple decision tree classifiers, have been widely used to detect nonlinear effects. RF offers high robustness and interpretability, not only accommodating high-dimensional datasets that are difficult for traditional regression models to analyze but also handling nonlinear relationships across different data types. GAMM incorporates smoothing functions to flexibly characterize nonlinear effects between independent and dependent variables and allows the inclusion of random effects to account for group- or area-level differences, thus combining interpretability with flexibility. In existing studies, researchers often used partial dependence plots (PDPs) from random forests and smoothing curves from GAMM to visually present the nonlinear effects of key variables and their inflection points. For example, Cheng et al. [29] employed a random forest model and used PDPs to visualize the complex relationship between the built environment and walking time among older adults. Wu et al. [30] applied GAMM and used smoothing curves to visualize the nonlinear effects of built environment features on walking frequency among older adults, identifying critical thresholds for factors such as population density, land-use mix, sidewalk density, and transit stop density.

The studies covered multiple spatial scales, including the city, street, and community levels (Fig. 3c). Regarding the choice of analytical units, studies at the community scale typically used grid cells or community buffers; those at the street scale often relied on block-level administrative boundaries; and studies at the city scale primarily employed city-level administrative units as the analytical framework.

### Built environment indicators influencing walking activity among older adults

In existing research, empirical investigations of the built environment’s impact on older adults’ walking activity have largely been framed by the “5D” model, encompassing density, diversity, design, destination accessibility, and distance to transit facilities [5]. To systematically organize the built environment measures used in previous studies and to provide a basis for subsequent scoring analyses, this review synthesized the built environment variables examined in the 46 included empirical studies, specifying their primary dimensions and detailed indicators (Table 3).

**Table 3:** Built environment indicators used in the 37 included empirical studies

Dimensions		Evaluation Indicator
Physical factors	Density	Population density (113), Residential density (55)
	Diversity	Land-use mix (137)
	Design	Intersection density (105), Green view index (48), Sidewalk density (48), Slope (26), Normalized Difference Vegetation Index (24), Proportion of green space (16), Green space area (16), Pedestrian crossing facilities (16), Street density (9), Walking path length (9), Sidewalk coverage ratio (9), Sidewalk connectivity (9), Number of bicycle lanes (9), Road network density (8), Link–Node Ratio (8), Building façade details (8), Forest coverage rate (8), Green coverage ratio (8), Sidewalk quality (8)
	Destination Accessibility	Distance to parks, green spaces, and plazas (39), Number of retail facilities (28), Number of educational facilities (25), Number of parks, green spaces, and plazas (25), fitness facilities (24), Number of medical facilities (23), Density of parks, green spaces, and plazas (17), Accessibility to parks, green spaces, and plazas (16), Density of recreational facilities (16), Accessibility to public service facilities (16), Number of food service facilities (16), Accessibility to commercial facilities (16), Distance to the nearest chess & card room (16), Number of recreational facilities (8), Accessibility to retail facilities (8), Distance to the nearest commercial center (8)
	Distance to Transit	Bus stop density (64), Number of bus stops (24), Accessibility to bus stops (23), Distance to the nearest bus stop (16), Number of bike-sharing stations (16), Bus line density (8), Distance to the nearest railway station (8), Distance to the nearest subway (8)
Perceived factors		Safety(71), Aesthetics༈47༉, Nighttime lighting༈15༉, Natural landscape comfort༈8༉

**Fig. 4:**
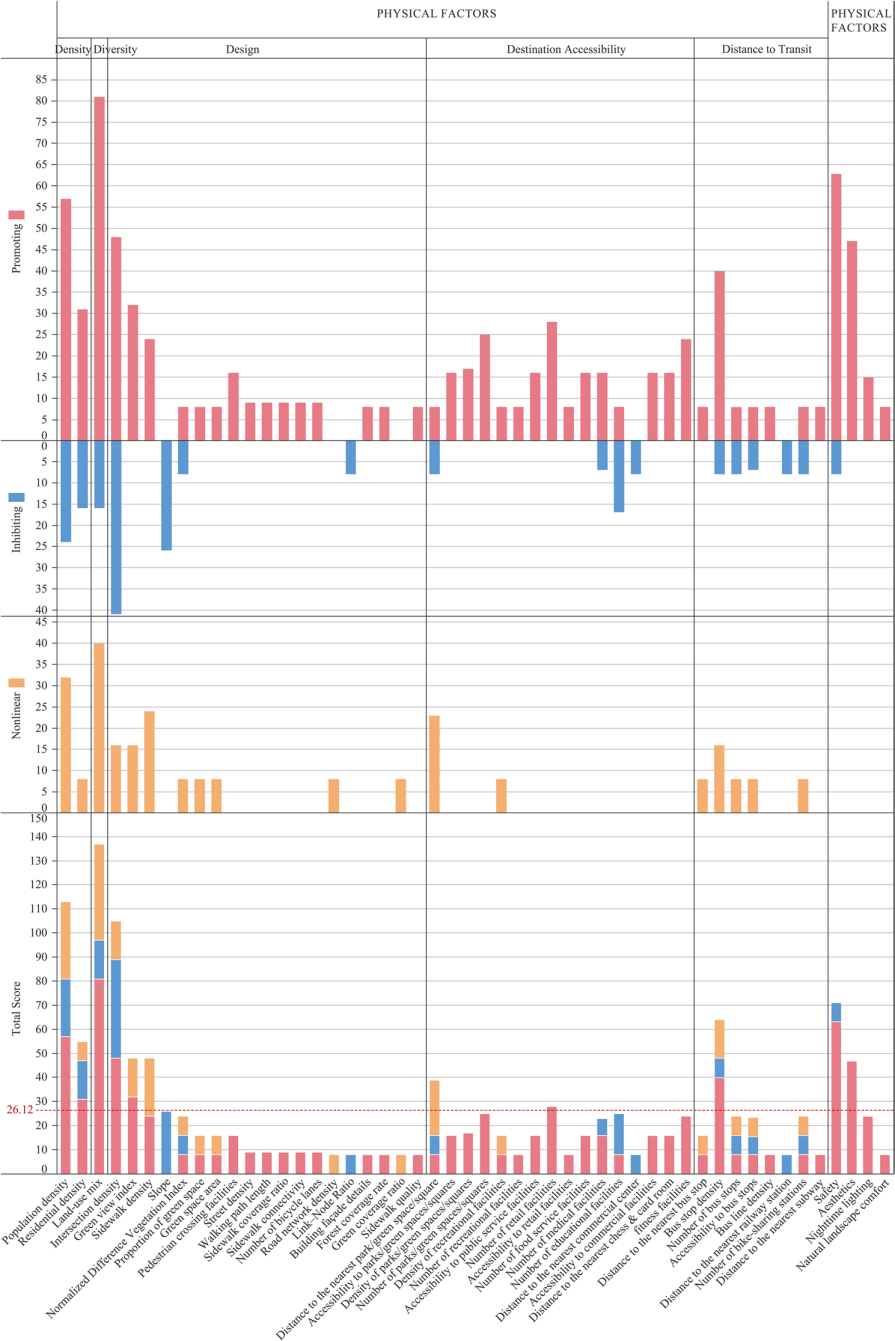
Visualization of Built Environment Indicators Assessed in the 37 Reviewed Studies

In this study, the quality of all included articles was assessed using the National Institutes of Health (NIH) Quality Assessment Tool for Observational Cohort and Cross-Sectional Studies, which consists of 14 items. For each article, a composite quality score was calculated based on item responses, with “Yes” scored as 1 and “No” scored as 0. The composite score was then used as the basis for weighting and assigned to the built environment factors examined in each study. For example, studies addressing population density were classified according to its reported effects on older adults’ walking activity, categorized as promotive, inhibitory, or nonlinear. The composite quality scores of studies within the same category were then aggregated to generate an overall score for the corresponding effect of each factor. Detailed quality scores of the included studies are provided in Appendix 2, and factor-specific scoring results are presented in Appendix 3. This approach enabled the quantification of built environment effects on older adults’ walking activity and facilitated the identification of key factors with stronger consensus. In presenting the results, cumulative scores were classified into promotive, inhibitory, nonlinear, and total categories. The overall mean score for built environment factors was 26.12, which was used to illustrate research attention and impact trends across factors (Fig. 4).

A total of nine physical factors and two perceived factors scored above the overall mean, reflecting greater attention and empirical support in the existing literature. The physical factors included land-use mix (137), population density (113), intersection density (105), transit stop density (64), residential density (55), green view index (48), sidewalk density (48), distance to parks/green spaces/plazas (39), and number of retail facilities (28). The perceived factors were safety (71) and aesthetics (47).

## Discussion

In examining the influence of built environment factors on older adults’ walking activity, existing studies have typically measured walking using indicators such as walking time, walking frequency, or walking propensity. To further reveal the mechanisms through which built environment elements affect walking behavior among older adults, this review selected key variables with composite scores above the overall mean. These variables were categorized into two groups: physical factors and perceived factors. Physical factors were further organized according to the 5Ds framework into five subcategories: density, diversity, design, destination accessibility, and distance to transit. Relevant subfigures were extracted from the included studies, and curve reconstruction and digitization were performed using the built-in image digitization tool of OriginPro 2024.

### Physical factors

#### Density

##### Population density

Most studies indicate that moderate to high population density helps increase walking frequency and duration. High-density communities are typically equipped with richer service facilities and compact spatial structures, which shorten travel distances and enhance older adults’ convenience and willingness to walk [18]. This promotive effect is particularly pronounced in areas with good walking accessibility [24]. However, this relationship is not uniformly positive. Excessively high population density is often accompanied by crowding, noise pollution, deteriorating air quality, and traffic safety risks, which undermine the comfort and sense of security of older adults when walking [19, 31].

**Fig. 5:**
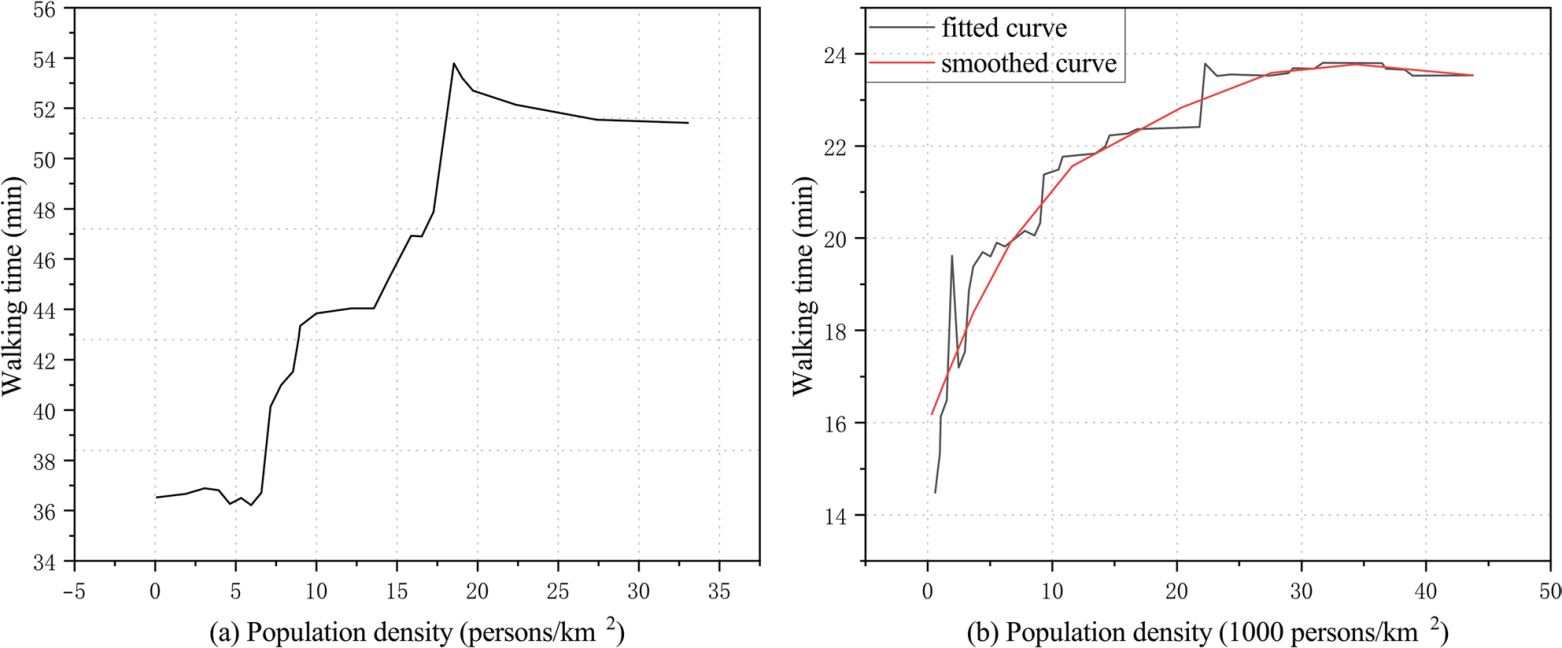
Relationship between population density and walking time among older adults. **a** Findings from Cheng et al. [32]: walking time increased significantly when population density rose from 6 to 20 persons/km^2^, but declined when density exceeded 20 persons/km^2^ due to crowding and safety pressures. **b** Findings from Wu et al. [33]: when population density exceeded 30,000 persons/km^2^, walking time plateaued at around 23 min and slightly decreased beyond 35,000 persons/km^2^ as environmental stress and congestion intensified

Cheng et al. found that when population density ranged from 6 to 20 persons/km^2^, walking time increased significantly, primarily because higher density environments improved infrastructure and service provision, thereby enhancing walking convenience. However, when density exceeded 20 persons/km^2^, crowding and safety pressures increased, posing threats particularly to older adults with weaker physical capacity and ultimately discouraging walking (Fig. 5a) [32]. Another empirical study reported that when population density exceeded 30,000 persons/km^2^, the average daily walking time of older adults plateaued at around 23 min. When density further increased beyond 35,000 persons/km^2^, walking time slightly declined (Fig. 5b) [33], likely due to intensified environmental stress and road congestion, which reduced walking comfort.

**Fig. 6:**
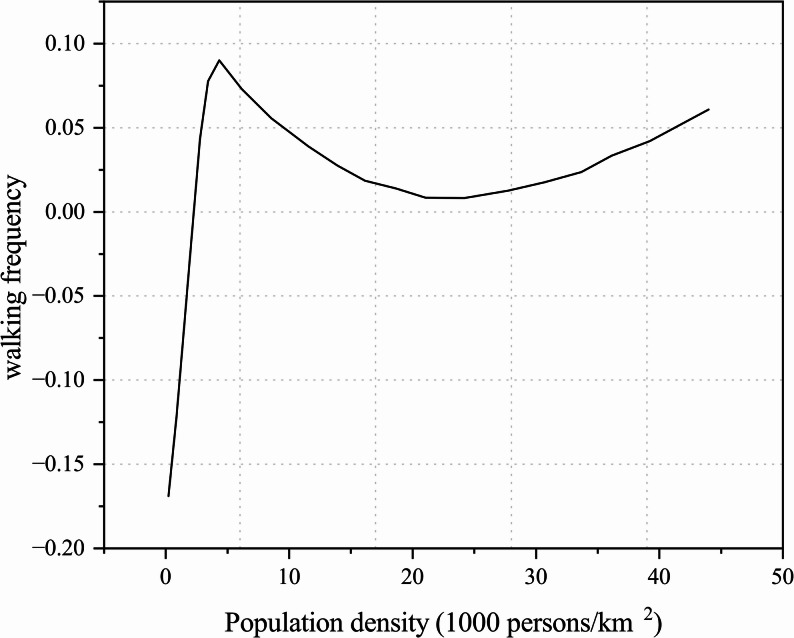
Relationship between population density and walking frequency among older adults

In a study using walking frequency as the dependent variable, walking frequency increased rapidly as population density rose to approximately 7,000 persons/km^2^, but beyond this threshold the marginal effect of density became negligible (Fig. 6) [34]. Although the concentration of functional facilities can enhance motivation for travel, excessive density may introduce disturbances and environmental complexity, thereby reducing the frequency of walking.

**Fig. 7:**
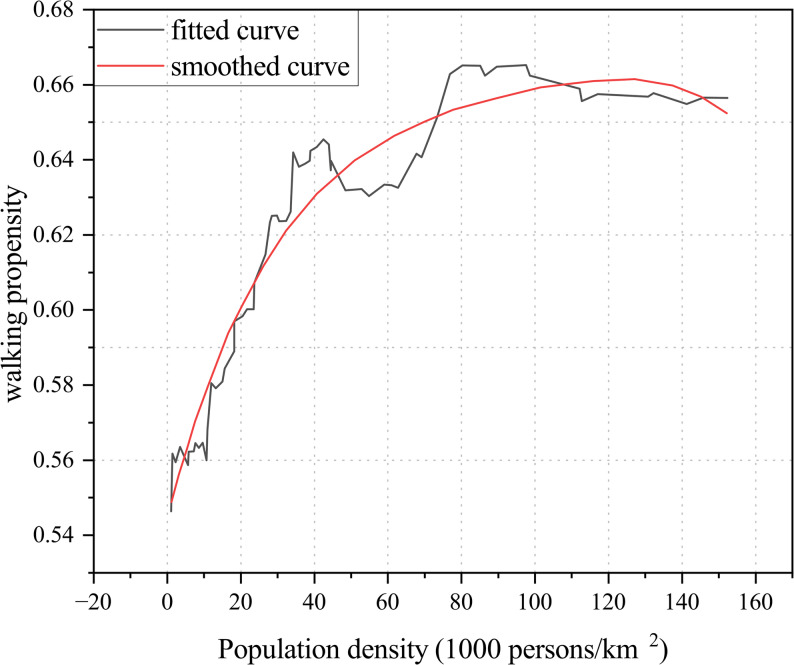
Relationship between population density and walking propensity among older adults

In studies examining older adults’ walking propensity—such as willingness to walk or preference for walking as the primary mode—population density showed a positive effect below 75,000 persons/km^2^ but turned negative beyond this threshold (Fig. 7) [35]. In high-density contexts, older adults may avoid walking due to safety concerns or perceived burdens, opting instead for alternative travel modes. Variations in sample sizes and measurement methods across studies contributed to substantial differences in results, suggesting the need for further research to validate the threshold of population density that promotes walking among older adults. In addition, a study by Cho et al. [36] on adult samples in Korea found that population density began to plateau and even turn negative within the range of 9,132–16,101 persons/km^2^, a threshold similar to that observed in studies of older adults.

In addition, the effects of population density varied substantially across different types of walking behavior. In a U.S. study [37], high-density communities significantly promoted utilitarian walking but had no notable impact on recreational walking. This may be attributed to the concentration of functional facilities and convenient transit access in high-density areas, which facilitate commuting and shopping-related walking, while limited recreational space and a sense of environmental stress constrain leisure walking.

Therefore, the mechanisms through which population density influences older adults’ walking activities are shaped by the combined effects of environmental quality, spatial structure, and travel purpose. Future research and urban planning should pay closer attention to the nonlinear effects of density, avoiding excessive density that creates environmental stress and discourages walking, while promoting health-oriented strategies for density optimization.

##### Residential density

Residential density, as an important indicator influencing older adults’ walking activity, exhibits distinct effects. In an empirical study conducted in Temuco, Chile, residential density was found to be significantly positively associated with older adults’ walking activity, with total walking time increasing as residential density rose [38]. This finding aligns with the compact city model, suggesting that in small- to medium-scale urban contexts, moderately increasing residential density can help promote walking activity. Higher residential density is often accompanied by more compact urban forms and greater land-use mix, thereby shortening the distance between residences and daily activity sites, improving destination accessibility, and reducing reliance on motorized travel [39, 40]. An analysis based on wearable device data in Changsha further demonstrated that higher residential density significantly increased walking frequency and distance among adults, with a stronger effect on walking than on running [41].

However, excessively high residential density may generate adverse effects. Studies have indicated that high-density communities often experience reduced per capita access to public green and recreational spaces, alongside environmental crowding, increased traffic pressure, and insufficient facility allocation. These factors collectively reduce older adults’ willingness to engage in daily walking within their communities [42]. When adequate green space, open areas, and walking-supportive facilities are lacking, high density may instead act as a barrier to walking among older adults [43].

**Fig. 8:**
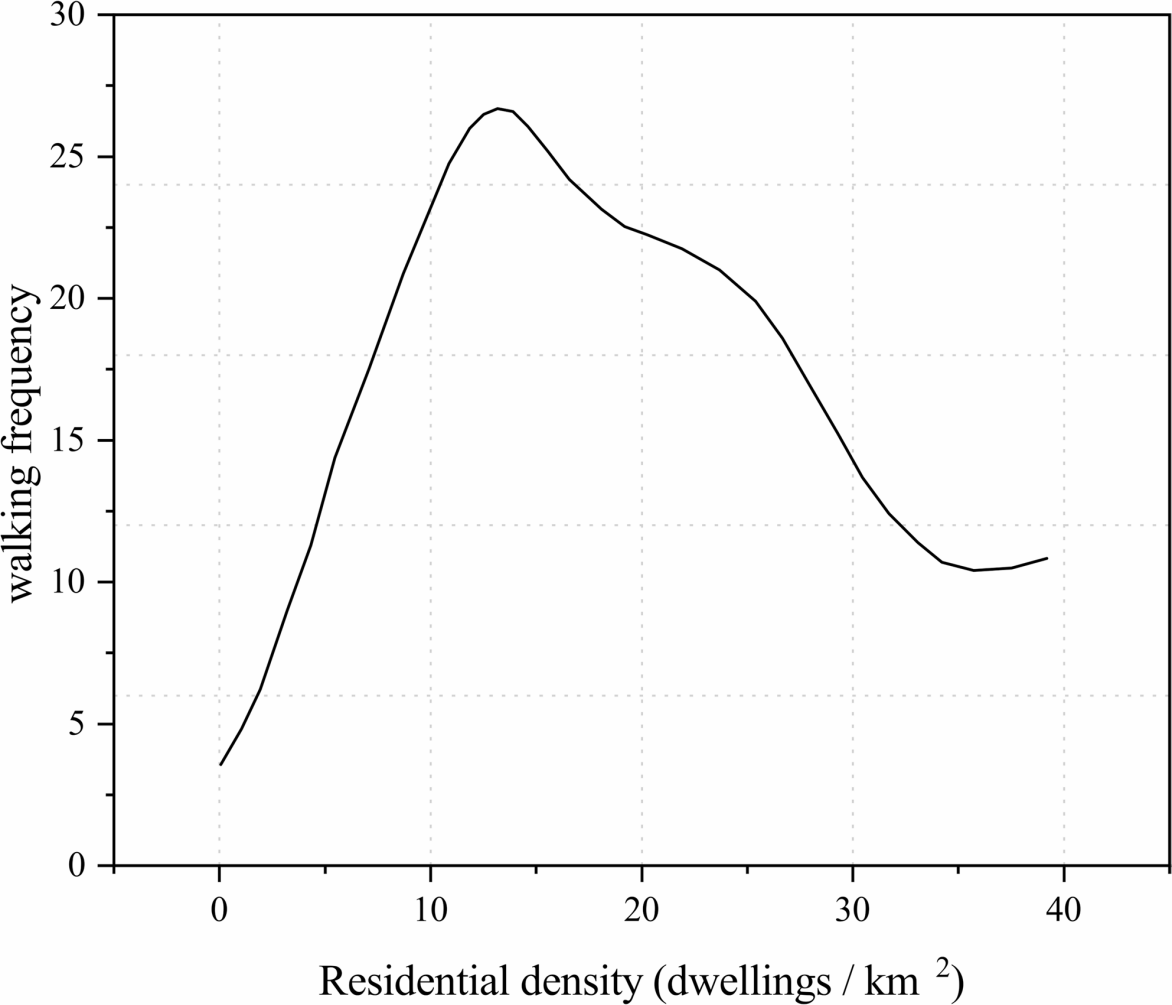
Relationship between residential density and walking frequency among older adults

In addition, the relationship between residential density and older adults’ walking activity is nonlinear. Cerin et al. found that at moderate density levels (approximately 10,000–25,000 households/km^2^), residential density had the strongest positive effect on walking frequency, providing sufficient destinations and transit accessibility without excessively compromising public space and environmental quality. Beyond this threshold, the positive effect gradually weakened and even turned negative, particularly for recreational walking, where it showed a clear inhibitory effect (Fig. 8) [25]. This is primarily because extremely high-density communities are often accompanied by limited green space, traffic noise, and spatial congestion, which collectively reduce older adults’ motivation to walk.

Therefore, for urban planning, this implies that in promoting compact development, it is essential to strike a balance between increasing density and maintaining livability, ensuring that residential density remains within a range that maximizes walking activity.

#### Diversity

##### Land-use mix

Higher land-use mix can effectively improve the convenience and purposefulness of daily walking among older adults, thereby enhancing their willingness to walk. In areas with high land-use mix, residential, retail, medical, and recreational facilities are reasonably located within walking distance, encouraging older adults to walk when meeting diverse needs such as shopping, healthcare, and social interactions [22]. Moreover, functionally diverse neighborhoods typically exhibit greater street vitality and pedestrian density, which not only increase the attractiveness and perceived safety of walking routes but also reduce feelings of loneliness and insecurity during travel among older adults. Evidence shows that older adults living in high land-use mix neighborhoods have significantly longer daily walking times and higher walking frequencies than those in single-function areas [20–45]. At the same time, the diverse destinations provided by land-use mix foster social interaction and community participation, further enhancing the attractiveness and actual prevalence of walking [20].

However, it is noteworthy that the impact of land-use mix on walking behavior does not always follow a linear positive trend; in some studies, nonlinear effects have also been observed.

**Fig. 9:**
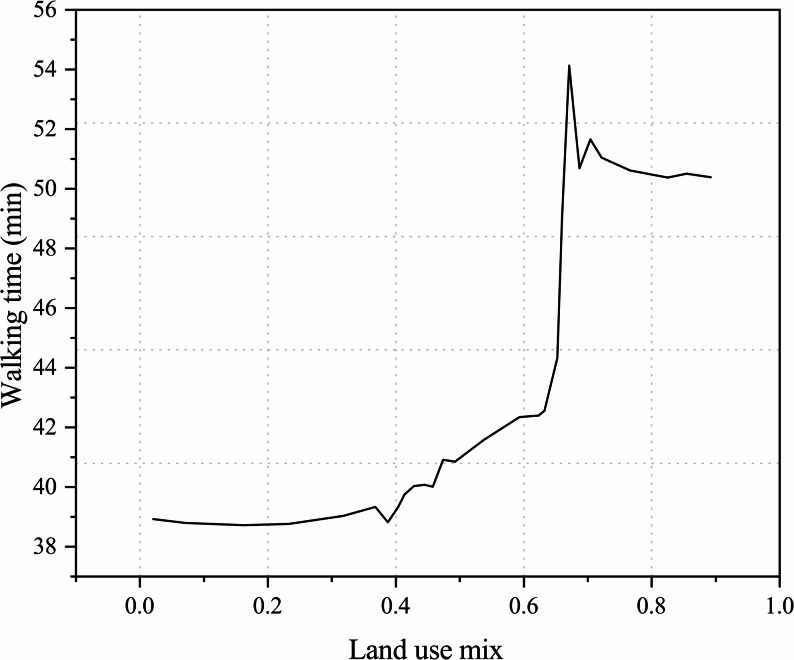
Relationship between land-use mix and walking time among older adults

In the study by Cheng et al., land-use mix was calculated based on five categories: residential, commercial, educational, recreational, and public services. The results showed an inverted U-shaped curve: walking time among older adults increased significantly as the land-use mix rose from 0.4 to 0.7, but the trend declined once the value exceeded 0.7.This “inverted U-shaped” curve may be explained by the high concentration of functions in areas with high land-use mix, where multiple travel tasks are consolidated, thereby reducing the total walking time (Fig. 9) [32].

**Fig. 10:**
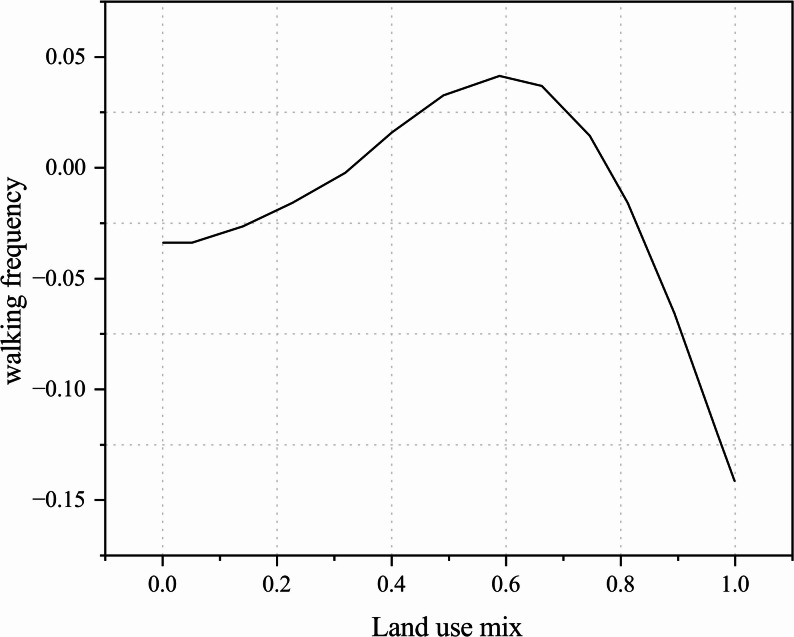
Relationship between land-use mix and walking frequency among older adults

Another study found that when the land-use mix index was below 0.65, walking frequency increased with higher levels of mix; however, beyond 0.65, excessive functional diversity may increase environmental complexity and perceived disturbances, thereby reducing walking frequency (Fig. 10) [34]. It is noteworthy that the land-use mix in this study was calculated using the entropy index (EI). Although the study did not provide detailed descriptions of the land-use categories, its findings were consistent with previous literature [46].

**Fig. 11:**
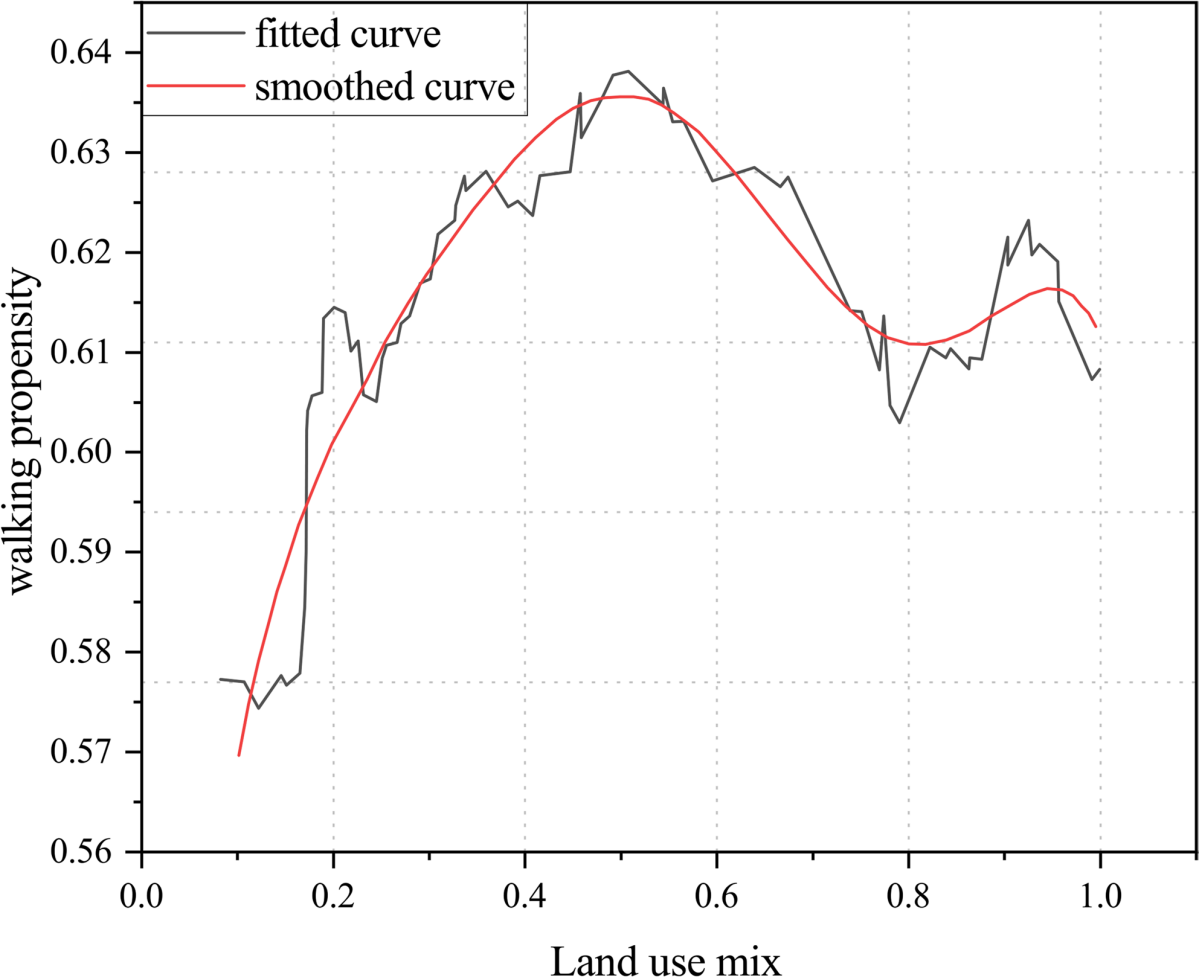
Relationship between land-use mix and walking propensity among older adults

In the study by Yang et al., the land-use mix index was calculated based on three categories: residential, office, and retail. The study found that when the land-use mix index was below 0.55, older adults’ walking propensity increased as the mix rose. However, within the 0.55–0.8 range, walking propensity declined with greater mix, while beyond 0.8, it again exhibited a positive trend (Fig. 11) [47].

Analysis of the relationship between land-use mix and older adults’ walking time, frequency, and propensity suggests that when the land-use mix exceeds the threshold of 0.7, it may suppress walking activity. A range of 0.55–0.7 is therefore recommended, with this nonlinear trend offering empirical guidance for urban land-use planning. However, as the types and numbers of land-use categories vary across studies, the results may involve some degree of bias, and future research should further validate these findings in light of regional characteristics.

In addition, empirical evidence suggests that when the land-use mix index exceeds 2.2, commuting-related walking increases significantly among middle-aged and younger adults. This discrepancy may reflect distinct behavioral logics across age groups in mixed-use environments: older adults rely more on simple, predictable settings and tend to achieve the best walking experience at moderate levels of mix, whereas middle-aged and younger adults—driven by frequent work and commuting demands, stronger environmental adaptability, and a preference for diverse destinations—benefit from highly mixed urban areas that provide more travel incentives and thereby reinforce walking behavior.

In summary, urban land-use configuration should adopt a moderate degree of mix, avoiding excessive functional diversity that creates environmental complexity and cognitive burden. Such balance can enhance neighborhood-level convenience and livability, thereby promoting walking activity and supporting the health of older adults.

#### Design

##### Intersection density

Higher intersection density increases route flexibility and optimizes travel efficiency, not only enhancing the convenience of walking for older adults but also strengthening neighborhood social interaction by providing more opportunities for engagement [48, 49]. Bonaccorsi et al. [22] noted that compact street structures with dense intersections facilitate older adults’ likelihood of engaging in daily strolling and non-commuting walking. However, some studies suggest that in contexts of high traffic density, complex road design, or inadequate pedestrian safety measures, excessive connectivity may increase the risk of traffic accidents—particularly for older adults crossing roads—thus exerting an inhibitory effect [50, 51].

**Fig. 12:**
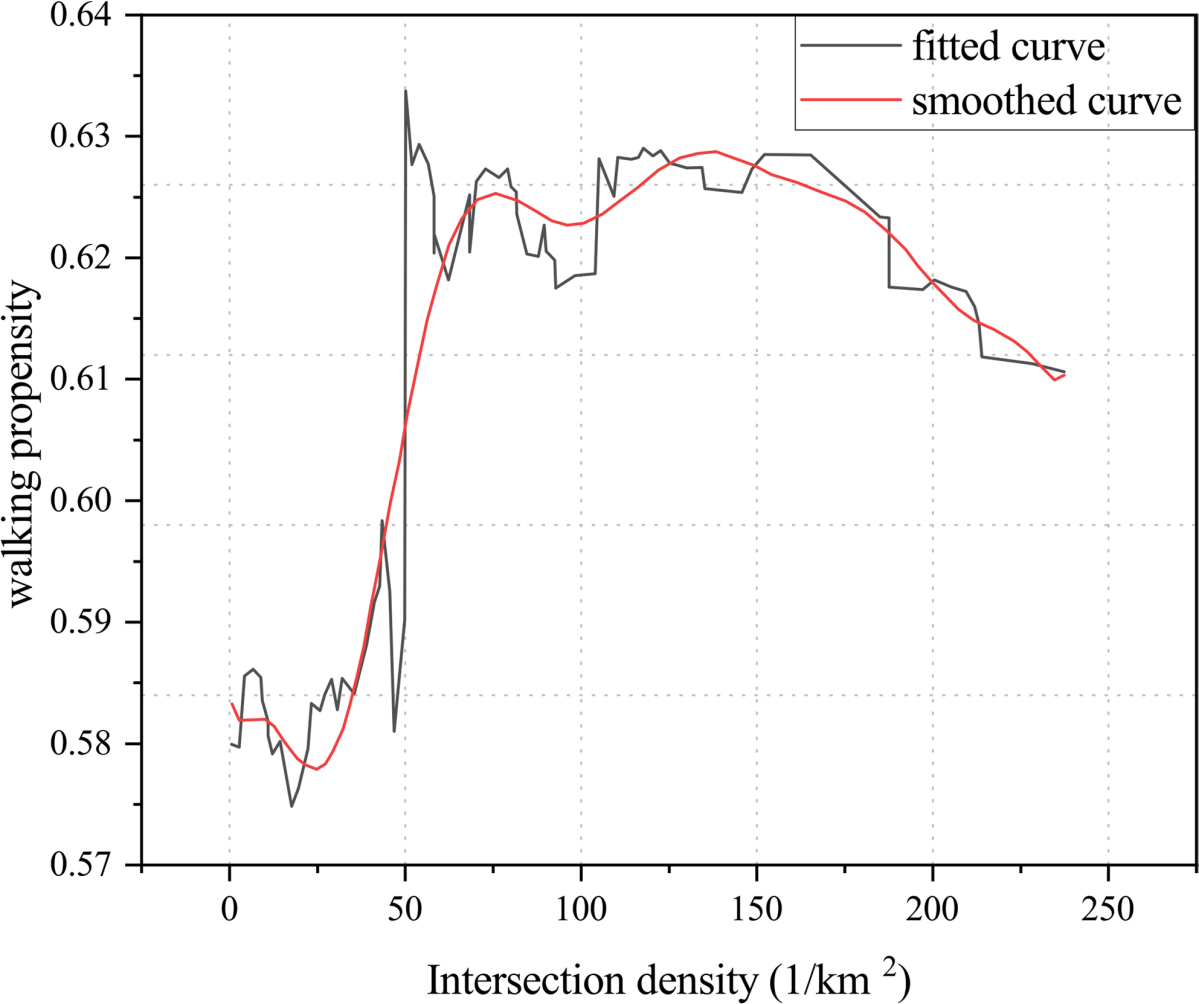
Relationship between intersection density and walking propensity among older adults

In addition, evidence shows a nonlinear relationship between intersection density and walking activity among older adults. A study in Luxembourg [40] found that street connectivity significantly increased walking probability at low to moderate levels (0–8 intersections), but beyond this threshold, the effect turned negative, further confirming the “threshold effect” of connectivity on walking behavior. Similarly, another study demonstrated that within the range of 25–65 intersections/km^2^, higher intersection density significantly promoted walking probability; however, beyond this range, its positive effect diminished and even turned inhibitory (Fig. 12) [47]. This suggests that moderate intersection density, by offering more direct routes and shorter distances to destinations, can enhance travel efficiency and effectively stimulate walking behavior. However, excessive connectivity may create route complexity and frequent traffic conflicts. In the absence of adequate safety infrastructure, this can cause disorientation and psychological discomfort, thereby discouraging walking among older adults.

By contrast, an empirical study on middle-aged and younger adults found that when the number of intersections was below 50, both commuting and leisure walking time were suppressed. However, once the number exceeded 100, leisure walking time increased significantly [52]. This divergence highlights differences in tolerance thresholds across age groups: older adults are more vulnerable to the safety risks and psychological burdens associated with excessive connectivity, whereas younger adults, with greater physical capacity, adaptability, and tolerance for complex environments, can still benefit from high connectivity in terms of leisure walking. Moreover, areas with dense intersections often coincide with commercial clustering and functional diversity, which attract younger populations through “shopping-oriented” walking, while older adults—prioritizing safety and simplicity—may experience reduced walking willingness in such settings.

Overall, the positive effects of intersection density must be grounded in safety and perceived comfort. Urban transport planning should balance compactness with walkability to prevent the negative consequences of excessive connectivity.

##### Green view index

The Green View Index (GVI), which quantifies the extent of vegetation visible at the street level, has gained increasing attention in recent years within healthy city and environmental behavior research. Moderate levels of street greenery enhance aesthetic appeal, regulate microclimates, and mitigate noise, thereby creating a more comfortable and psychologically supportive walking environment for older adults. These improvements significantly increase both walking frequency and duration [48]. Empirical evidence further shows that when streets feature high levels of vegetation on both sides, older adults report stronger feelings of safety and enjoyment. This effect is particularly pronounced in urban contexts with high temperatures, heavy traffic, or visually monotonous environments, where greenery alleviates anxiety and encourages walking [53, 54].

**Fig. 13:**
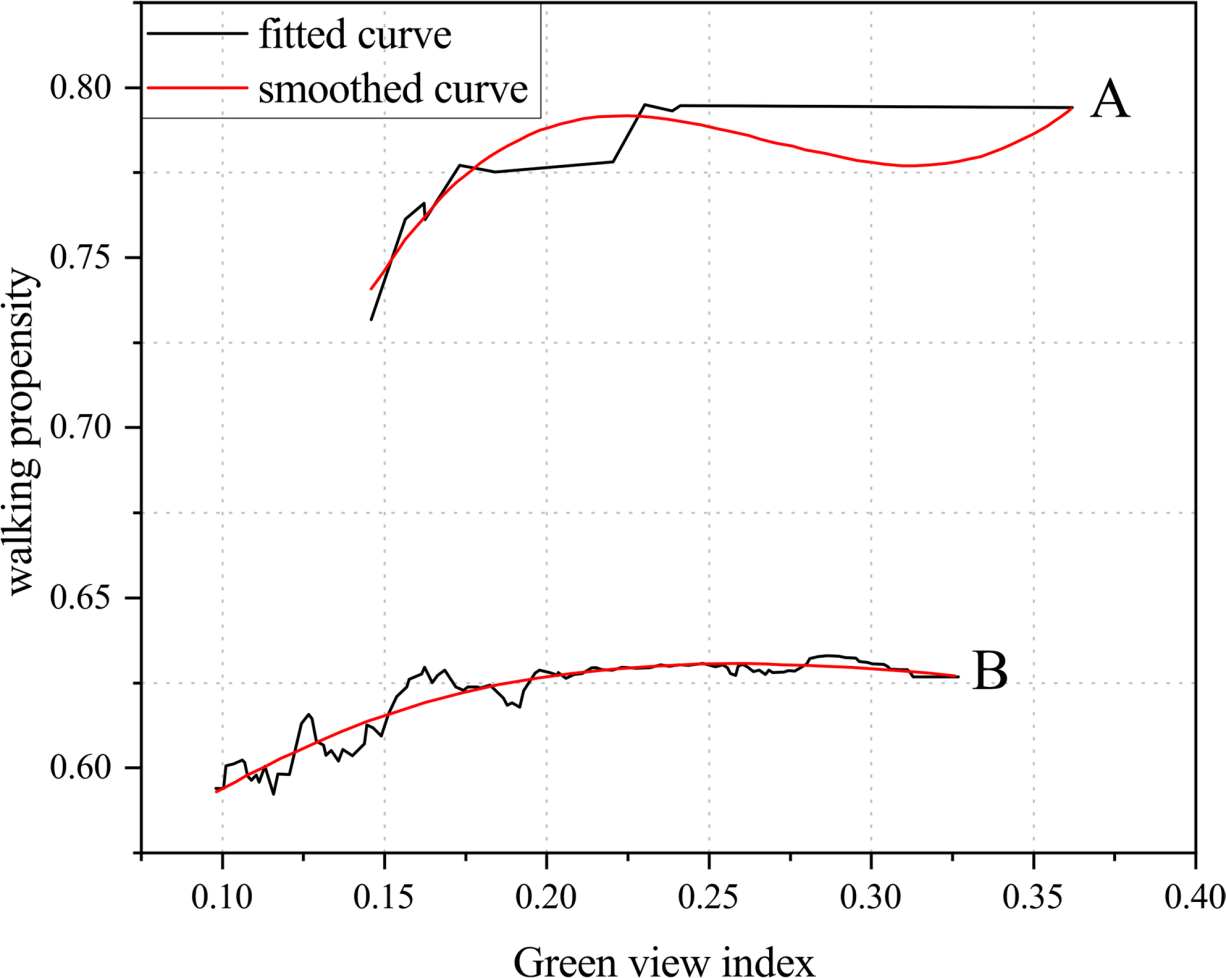
Relationship between streetscape green view index (GVI) and walking propensity among older adults. **A** Findings from Zang et al. [55]: walking propensity increased with GVI up to approximately 0.24, after which it declined, indicating a threshold effect of streetscape greenery. **B** Findings from Yang et al. [35]: walking willingness decreased once GVI exceeded 0.24, likely due to excessive vegetation reducing visibility and spatial recognition

However, the influence of GVI on walking behavior among older adults is not linearly increasing. Beyond a certain threshold, its marginal benefits diminish and may even turn negative in some contexts. One study found that predicted walking propensity peaked when GVI reached approximately 0.24, after which it declined, consistent with other findings. This suggests that the positive effects of streetscape greenery have a threshold: once GVI exceeds 0.24, further increases no longer encourage walking and may reduce its attractiveness due to limited functionality or reduced visual permeability (Fig. 13A) [55]. Similarly, Yang et al. reported that walking willingness decreased once GVI surpassed 0.24 (Fig. 13B) [35]. Possible explanations include excessive vegetation obstructing sightlines, blurring spatial boundaries, and complicating path recognition, which in turn undermine perceptions of safety and spatial control. In addition, although dense greenery provides shade and cooling, it may reduce visibility in winter or low-light conditions, thereby discouraging walking.

Therefore, urban greenery design should balance visibility and functionality, avoiding streetscapes that become monotonous “green tunnels” with visual redundancy and unclear orientation. Planting schemes should go beyond maximizing coverage, integrating street width, pedestrian flow, and spatial layout to create environments with visual hierarchy and directional clarity. Such design not only enhances wayfinding but also provides psychological support. Evidence suggests that when the Green View Index (GVI) exceeds 0.24, walking willingness may decline. This highlights the importance of considering the 0.24 threshold in urban planning and future research to improve the safety and comfort of walking environments for older adults.

##### Sidewalk density

Sidewalk density has been consistently shown in empirical studies to be positively associated with walking activity among older adults. Higher sidewalk density indicates a more continuous pedestrian network and better supporting facilities, which reduce inconveniences and potential risks during travel, thereby creating a safer and more comfortable walking environment for older adults [56, 57]. In addition, dense and connected sidewalk systems provide more diverse and direct route options, shorten travel distances to destinations, and enhance both walking efficiency and willingness, ultimately promoting walking behavior [58].

**Fig. 14:**
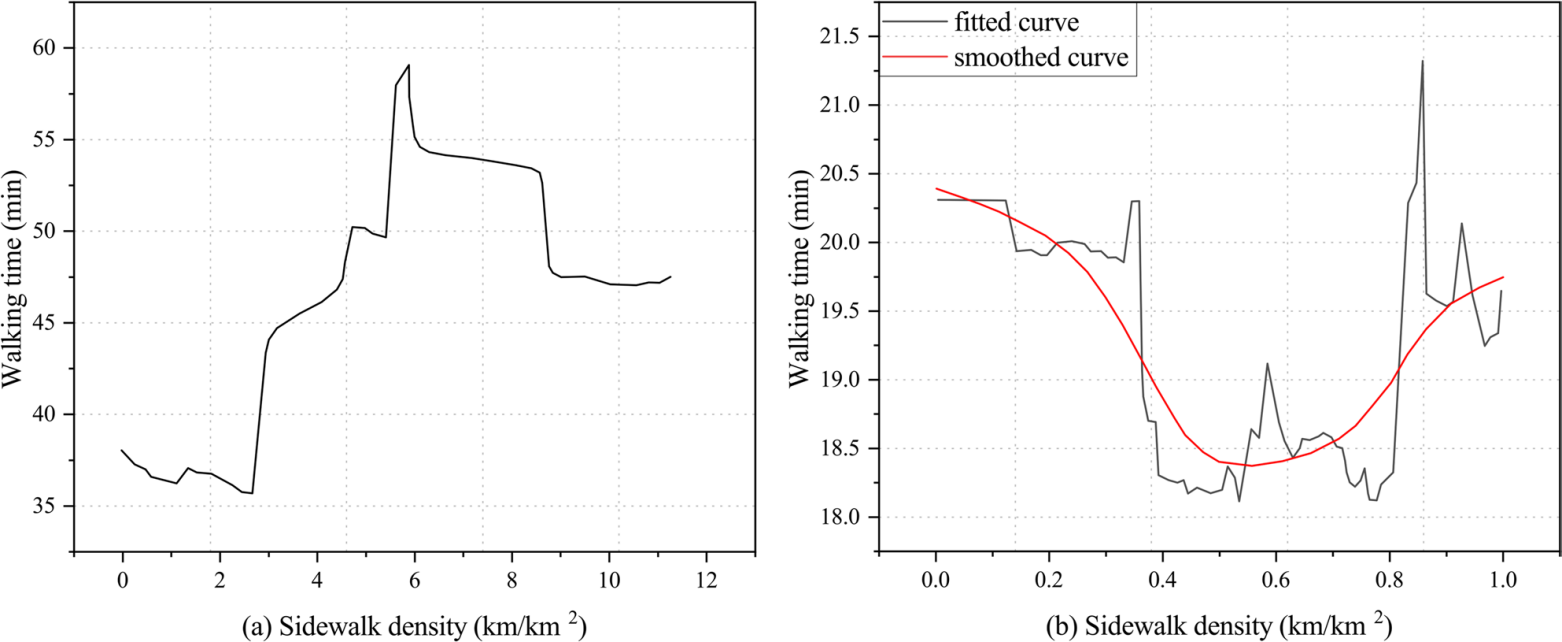
Relationship between sidewalk density and walking time among older adults. **a** Findings from Cheng et al. [29]: an inverted U-shaped association was observed; walking duration increased when sidewalk density was below 6 km/km^2^ but declined when it exceeded this threshold, reflecting potential safety risks and psychological burdens from overly complex networks. **b** Findings from Wu et al. [59]: a positive U-shaped relationship was found; when sidewalk density was between 0 and 6 km/km^2^, its effect on walking time was negative, but walking duration increased once density surpassed 6 km/km^2^, with the most pronounced gains within the 6–12 km/km^2^ range

However, several studies indicate that the relationship between sidewalk density and walking is not strictly linear but instead exhibits distinct nonlinear patterns. Cheng et al. reported an inverted U-shaped association: when sidewalk density was below 6 km/km^2^, walking duration increased significantly, suggesting that continuous and accessible pedestrian networks promote walking among older adults. Yet, once this threshold was exceeded, walking duration declined, reflecting the potential safety risks and psychological burdens posed by overly complex street networks and frequent intersections, which reduce the overall attractiveness of the walking environment (Fig. 14a) [29]. In contrast, Wu et al. identified a positive U-shaped pattern: when sidewalk density ranged between 0 and 6 km/km^2^, its effect on walking duration was negative; however, once density surpassed approximately 6 km/km^2^, walking duration increased, with the most pronounced gains observed within the 6–12 km/km^2^ range, where average walking time rose from 18.3 to 20.3 min (Fig. 14b) [59].

Notably, both studies identified 6 km/km^2^ as a critical turning point in sidewalk density. Cheng et al. cautioned that this threshold should be interpreted carefully due to limited data, whereas Wu et al. argued that higher sidewalk density can provide more diverse walking routes and foster a walkable environment characterized by strong street connectivity. This finding is broadly consistent with the baseline sidewalk density recommended in China’s national design standards (6–10 km/km^2^) [60]. Accordingly, future research should further focus on the 6 km/km^2^ sidewalk-density threshold to test its generalizability and applicability.

Taken together, in highly connected street networks, frequent intersections and complex road geometries may increase older adults’ crossing burden and traffic-safety risk, thereby discouraging walking. Therefore, urban planning and street design should avoid simply maximizing connectivity and instead prioritize intersection safety and accessibility, maintaining access while reducing network complexity to create walking environments that are more comfortable, continuous, and safe.

#### Destination accessibility

##### Distance to parks, green spaces, and plazas

Greater distances to parks significantly reduce the likelihood of walking among older adults. Empirical evidence shows that parks requiring more than 10 min of walking are often perceived as inaccessible, thereby lowering the willingness for active travel [61]. A systematic review by Bancroft et al. in U.S. cities also reported that for every 500-meter increase in park distance, the frequency of park-related walking among older adults decreased by an average of 13% [62]. This effect is particularly pronounced among the very old and those with chronic conditions.

**Fig. 15:**
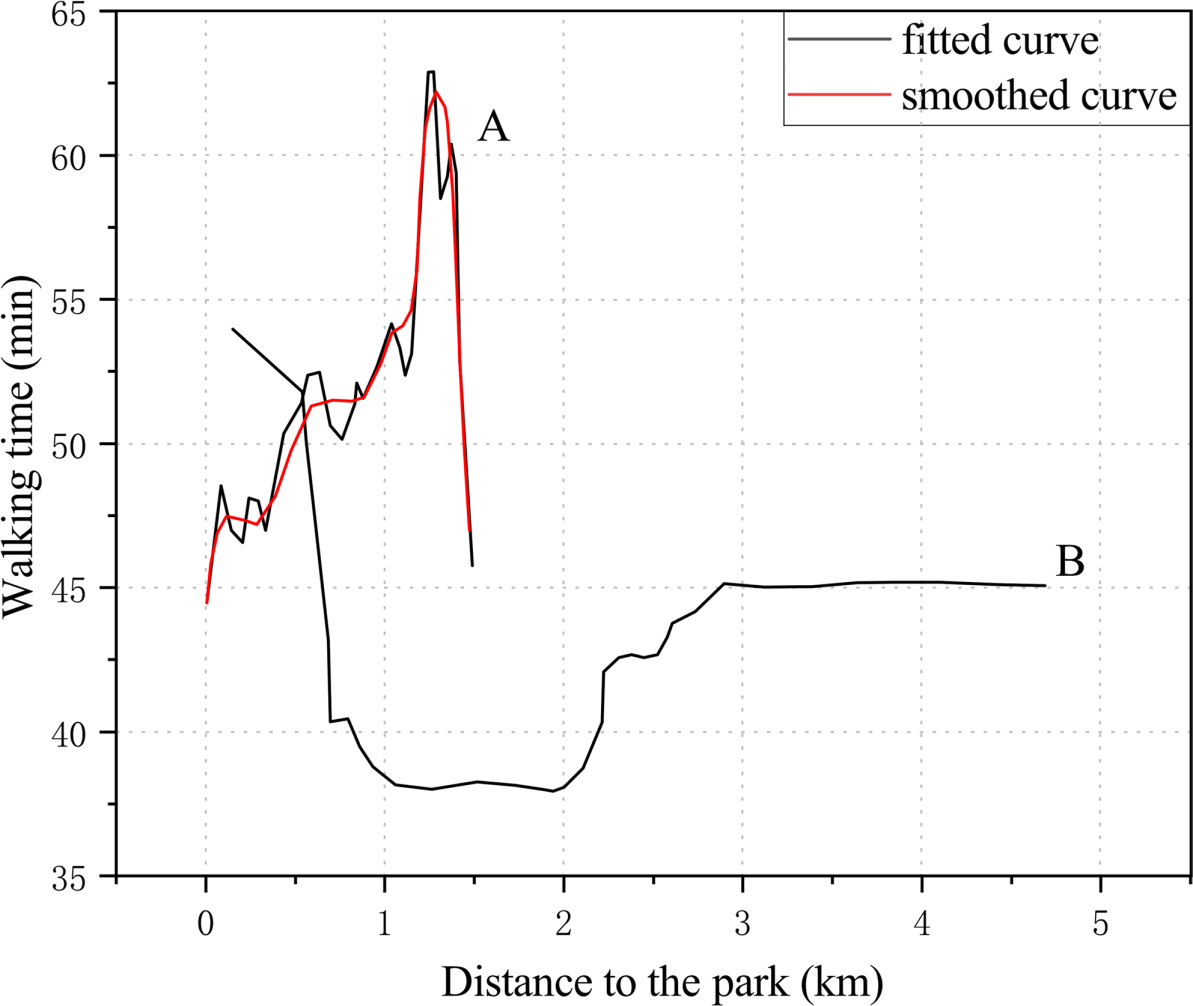
Relationship between distance to parks and walking time among older adults. **A** Findings from Zhu et al. [63]: when park distance ranged from 0 to 1.3 km, walking time increased with distance but declined significantly beyond 1.3 km. **B** Findings from Cheng et al. [32]: significant effects were observed within the 0–1 km and 2–4.3 km ranges; walking time was negatively correlated with distance below 1 km but increased between 2 and 3 km. Beyond 3 km, the facilitative effect was no longer significant

However, the relationship between distance to parks and walking activity is not strictly linear. Zhu et al. found that when park distance ranged from 0 to 1.3 km, walking duration increased with distance, but beyond this range it declined significantly. This suggests that approximately 1.3 km may represent the maximum acceptable walking distance for older adults; parks located farther away lack attractiveness due to limited accessibility (Fig. 15A) [63]. In addition, Cheng et al. reported reliable effects only within the 0–1 km and 2–4.3 km distance ranges. When distance was less than 1 km, park distance was significantly and negatively correlated with walking duration—residing farther from parks reduced the likelihood of walking, consistent with prior findings [64, 65]. Interestingly, when distance increased from 2 to 3 km, walking duration rose instead, possibly because older adults in this range intentionally treated park visits as opportunities for exercise and recreation. However, once distance exceeded 3 km—beyond the scope of daily activities—its facilitative effect on walking was no longer significant (Fig. 15B) [32].

Based on the overall evidence, Cheng et al.’s view that “longer distances reduce daily walking among older adults” is more convincing and agrees with most empirical findings. In contrast, Zhu et al.’s study may reflect older adults’ intentional trips to parks rather than casual daily walking, which partly matches Cheng et al.’s observation of purposeful visits within the 2–3 km range. Future studies could focus on accessibility within 1 km to examine differences and tendencies in older adults’ walking behavior, offering more practical guidance for green space planning in age-friendly communities.

Overall, urban planners should make sure that residents have good access to parks within 1 km of their homes, so as to maximize their role in encouraging walking.

##### Number of retail facilities

Studies have found a significant positive association between the number of retail facilities and the walking levels of older adults. Older adults living in communities with local retail centers walk, on average, about one hour more per week than those in communities without such facilities. This effect remains significant even after controlling for health status, social relationships, and other individual characteristics, suggesting that retail facilities themselves are key drivers of walking behavior [66]. Similarly, Jia et al. [67] confirmed that the number of retail stores is the most influential built environment factor affecting walking activity among older adults. Older adults often lack motivation to walk solely for exercise, but routine activities such as shopping provide a practical reason for walking, thereby integrating physical activity naturally into their daily lives. In addition, the clustering of retail facilities may enhance community vitality and perceived safety. Vibrant commercial activity is usually accompanied by higher pedestrian traffic and greater street-level activity, which can help alleviate feelings of loneliness and reduce perceived risks while walking among older adults [68].

These findings suggest that age-friendly urban planning should prioritize the rational distribution and provision of retail facilities. Establishing sufficient small shops and convenience services within communities can enable older adults to achieve frequent, short-distance walking in their daily routines.

#### Distance to transit

##### Bus stop density

It is widely recognized that the “walk–ride–walk” trip chain of public transit systems relies on pedestrian access. An increase in the number of transit stops not only expands travel opportunities but also indirectly stimulates older adults to walk more frequently and for longer durations [67]. Dense transit networks are often associated with better road accessibility and facility proximity, which in turn enhance perceived safety and community familiarity [69]. At the same time, supporting infrastructure such as bus shelters, sidewalks, and slow-traffic systems provides older adults with a safer and more comfortable walking environment [70].

**Fig. 16:**
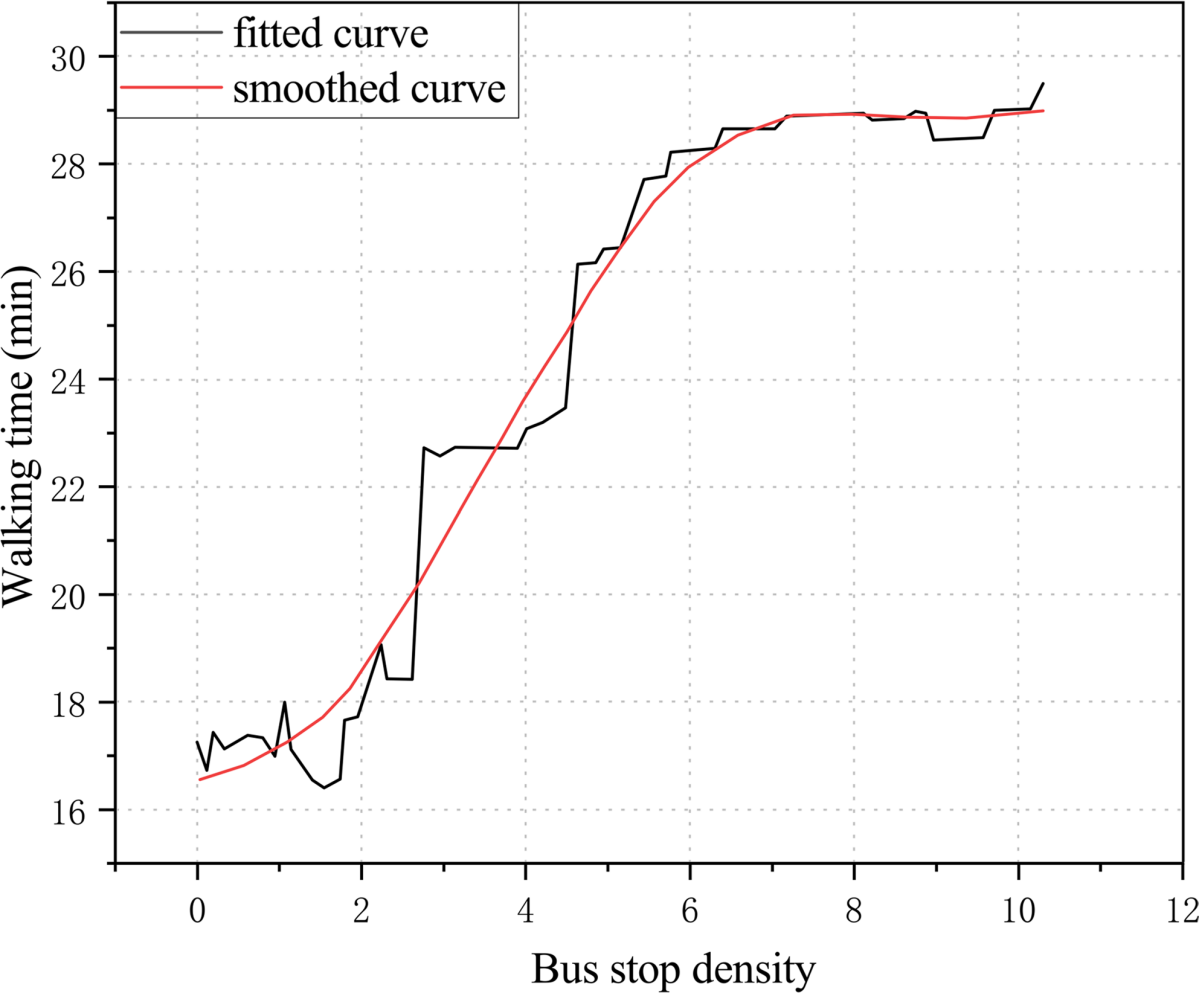
Relationship between transit stop density and walking time among older adults

Nonlinear analyses have revealed a threshold effect in the influence of transit stops on walking behavior. Wu et al. reported that as stop density increased to 7 per km^2^, average walking time rose from 16 to 28 min. Beyond this point, however, the effect plateaued, indicating a typical saturation effect (Fig. 16) [33].

Another study also found that when transit stop density was below 6 per km^2^, it was positively associated with walking frequency. However, once this threshold was exceeded, the promoting effect began to level off (Fig. 16) [34]. This phenomenon suggests that in the early stages of density increase, transit networks can shorten access distances and enhance public transport accessibility, thereby motivating older adults to walk as a mode of access. However, when stops become overly dense, spatial coverage largely meets travel needs, and adding more stops no longer sustains additional motivation for walking.

In summary, the influence of transit stop density on older adults’ walking behavior follows a nonlinear pattern, with significant increases in low-density ranges and saturation effects at higher densities. This finding indicates that urban transit planning should strategically maintain stop density within the optimal range of 6–7 stops per km^2^ to maximize the walking incentive effect.

### Perceived factors

#### Safety

A growing body of empirical evidence indicates that perceived safety plays a decisive role in shaping older adults’ walking behavior. First, traffic safety is closely associated with both walking duration and frequency. Liu et al. [71] found that older adults who were satisfied with sidewalk conditions and traffic safety were more likely to extend their walking time. Similarly, Tsunoda et al. [72], in a study conducted in rural Japan, demonstrated that a strong sense of traffic safety significantly increased the likelihood of older adults engaging in high levels of walking, highlighting that a safe road environment is a core condition for promoting daily walking. Second, perceived neighborhood safety is also an important determinant of recreational walking among older adults. In a study from Birmingham, United Kingdom, older adults living in high-poverty neighborhoods generally reported poorer perceptions of safety, which contributed to a marked reduction in their outdoor walking levels [73]. Similarly, a study from Brazil found that older adults who perceived higher levels of community safety during the day were more likely to engage in recreational walking, and that social support further strengthened this association [74].

However, a study conducted in Hong Kong revealed an opposite conclusion: perceived safety was negatively associated with walking levels among older adults [39]. The researchers suggested that this may be explained by the fact that communities with lower socioeconomic status often exhibit both poorer safety conditions and lower rates of motor vehicle ownership. As a result, residents rely more heavily on walking and public transit for daily travel, leading to higher levels of walking observed in less secure neighborhoods.

This phenomenon can also be interpreted in line with Maslow’s hierarchy of needs. Safety needs, as the foundational level immediately above physiological needs, constitute a prerequisite for older adults to engage in any outdoor activity. Only when traffic and neighborhood safety are ensured can older adults adopt walking as a vital means of maintaining health, fostering social interactions, and even pursuing self-actualization. Conversely, when a sense of safety is lacking, walking may be restricted or forced to occur in unfavorable contexts. This not only reduces physical activity levels but may also diminish opportunities for fulfilling higher-order needs.

In summary, older adults’ perception of safety directly influences both the initiation and continuation of walking. Urban planning and intervention strategies should urgently prioritize measures such as improving traffic safety infrastructure, strengthening security management, and enhancing nighttime lighting to optimize walking environments for older adults.

#### Aesthetics

An aesthetically pleasing neighborhood environment can effectively stimulate voluntary and recreational walking among older adults. A positive visual experience not only creates a sense of safety and enjoyment but also significantly enhances older adults’ feelings of belonging and trust in public spaces, thereby increasing their willingness to go out and their walking frequency [75, 76]. Aesthetic quality often interacts with other perceived factors—such as pedestrian space integrity, street maintenance, and neighborhood sociability—to create a pleasant travel environment. Studies have shown that aesthetically appealing neighborhoods also tend to feature well-defined pedestrian spaces, better street maintenance, and higher levels of social friendliness, where older adults demonstrate significantly greater walking duration and frequency. This suggests that aesthetic quality stimulates walking not only through “visual incentives” but also indirectly promotes physical activity by enhancing neighborhood belonging and perceived safety [22].

However, when aesthetic quality is modeled together with variables such as green view index, street connectivity, or environmental noise, its independent effect may become nonsignificant. This suggests that the influence of aesthetics is complex and largely dependent on multivariable interaction mechanisms. For example, Li et al. [77] found that in areas with poor public transport accessibility, aesthetic quality had a stronger motivating effect on walking behavior, whereas in function-oriented, commuter-dominated neighborhoods, its influence was relatively weakened. Therefore, although aesthetic quality is not the sole direct driver of walking, it plays an important indirect role in encouraging recreational and prolonged walking among older adults by enhancing perceived environmental quality and psychological safety.

### Limitations & future research recommendations

This study also has certain limitations. First, with the exception of one longitudinal study, most of the studies included in this review employed cross-sectional designs, making it difficult to establish causal relationships between the built environment and walking activity among older adults. Cross-sectional studies are often unable to adequately control for time-varying factors and cannot capture the lagged effects of policy or environmental modifications. In addition, if surveys are conducted within a specific season, seasonal bias may also be introduced.

Second, most of the studies included in this review did not adequately control for residential self-selection bias. Residential self-selection bias refers to the phenomenon in which individuals choose their place of residence based on travel tendencies, preferences, and socioeconomic status. For example, an older adult with a positive attitude toward walking and a preference for strolling may choose to move to a neighborhood that is walkable and supportive of walking. Therefore, the observed associations between the environment and walking behavior may reflect individual lifestyle preferences rather than direct causal relationships, potentially leading to an overestimation of built environment effects.

Third, inconsistencies in walking measures, sample sizes, and research methods across studies mean that the generalization and interpretation of threshold effects often remain speculative and somewhat subjective. Moreover, the visualization of threshold effect results in existing studies remains limited and requires improvement through more rigorous and scientific techniques.

Fourth, the geographic distribution of the included studies is relatively concentrated, with most conducted in China and the United States, which limits the generalizability of the findings to other sociocultural and urban contexts. Differences across countries in urban form, transportation systems, sociocultural contexts, and travel habits may lead to substantial variation in how built environment factors influence walking activity among older adults.

For future research, first, strengthening investigations into the nonlinear relationships between the built environment and older adults’ walking activities may help to uncover underlying mechanisms, avoid the limitations of linear assumptions, and provide evidence for developing more targeted health promotion strategies. Second, research is shifting from macro-level environmental factors to the micro-level of older adults’ subjective experiences, focusing on more nuanced aspects such as psychological perceptions and spatial cognition [78]. Refined analyses of green spaces, sedentary behavior, and daily functional capacity are gradually emerging as new research frontiers. Future studies should further conduct fine-grained analyses around individual perceptions and micro-mechanisms to improve the specificity and effectiveness of interventions. Finally, the integration of machine learning and artificial intelligence with big data offers broad prospects for predicting walking behavior and modeling its mechanisms. Future research could employ ensemble learning methods such as Random Forest and XGBoost, as well as deep learning approaches including RNNs and LSTMs, to construct dynamic prediction models of walking behavior in older adults [79–82]. These models can help uncover relationships between the built environment and walking activity, providing technical support for refined spatial interventions.

## Conclusions

This study systematically reviewed 46 empirical studies to comprehensively examine the influence of built environment characteristics on older adults’ walking activity and its potential mechanisms The results indicate that, among physical factors, the number of retail stores, and among perceptual factors, safety and aesthetic quality, play positive roles in significantly enhancing walking levels among older adults. Meanwhile, factors such as population density, residential density, land-use mix, intersection density, streetscape greenery, sidewalk density, distance to parks, and transit stop density all exhibited nonlinear effects.

Specifically, findings regarding population density varied considerably across studies, suggesting that future research should aim to verify the optimal threshold for population density. Residential density between 10,000 and 25,000 households per km^2^ had the strongest positive effect on walking among older adults. Land-use mix enhances destination diversity and strengthens travel motivation and flexibility. However, when the index exceeds 0.7, excessive mixing may increase cognitive burden, weaken environmental perception, and suppress walking willingness and frequency. It is recommended that land-use mix be maintained within the range of 0.55–0.7 to more effectively promote walking activity among older adults. Intersection density between 25 and 65 per km^2^ significantly promotes walking among older adults. Streetscape greenery exhibits an asymmetric trend, initially increasing and then leveling off or even declining. A level of around 24% is suggested to enhance visual appeal while balancing greenery with accessibility and openness. Future studies should pay particular attention to the threshold of 6 km/km^2^ in sidewalk density for further investigation. The acceptable maximum walking distance to parks, green spaces, or plazas is about 1–1.3 km. It is recommended that highly accessible green spaces be prioritized within a 1 km walking radius to maximize their benefits. Sufficient small shops and convenience facilities should be provided within communities to encourage walking among older adults. Transit stop density should be maintained within the reasonable range of 6–7 per km^2^ to maximize the incentive effect on walking. Although safety and aesthetic quality are not direct determinants of walking, they play crucial indirect roles in promoting walking among older adults by enhancing perceived environmental quality and psychological safety.

The findings are also consistent with the World Health Organization’s (WHO) core concept of “age-friendly cities.” For example, in terms of outdoor spaces and buildings, threshold indicators can be used to guide the improvement of walking environments; in transportation, they can inform the evaluation of road network design; and in social participation, improved walkability can facilitate daily travel and social interaction among older adults. In addition, the findings contribute to the development of age-friendly communities by supporting older adults’ social participation and quality of life, thereby providing both theoretical foundations and practical guidance for human-centered, age-friendly urban design.

## Supplementary Information

Supplementary Material 1.

Supplementary Material 2.

Supplementary Material 3.

## Data Availability

All data generated or analysed during this study are included in this published article and its supplementary information files.
