# Supplementary material for: Systematic review of nonlinear associations between the built environment and walking in older adults
Source: BMC Public Health. 2025 Dec 11;25:4240. doi: 10.1186/s12889-025-25141-6 (PMC12696931; doi:10.1186/s12889-025-25141-6)
Supplement: Supplementary file 1 — Supplementary Material 1. [file 12889_2025_25141_MOESM1_ESM.docx]

**Supplementary Table S1.** Search strategies for MEDLINE, PubMed, Web of Science, Scopus, and Embase

| **Database** | **Serial No.** | **Search contents** |
| --- | --- | --- |
| MEDLINE | #1 | built environment* OR urban environment OR environment design OR urban design OR neighborhood* OR pedestrian environment OR street OR physical environment OR walkability OR residential environment OR community environment |
|  | #2 | walking activity OR physical activity OR mobility OR walking behavior |
|  | #3 | older adult* OR elderly OR seniors OR aged OR older people OR older persons OR older citizens OR late life |
|  | #4 | #1 AND #2 AND #3 |
|  | Full search string | ((built environment* OR urban environment OR environment design OR urban design OR neighborhood* OR pedestrian environment OR street OR physical environment OR walkability OR residential environment OR community environment) AND (walking activity OR physical activity OR mobility OR walking behavior)) AND (older adult* OR elderly OR seniors OR aged OR older people OR older persons OR older citizens OR late life) AND ((medline[Filter]) AND (classicalarticle[Filter] OR comparativestudy[Filter] OR evaluationstudy[Filter] OR multicenterstudy[Filter]) AND (humans[Filter]) AND (english[Filter]) AND (1964:2024[pdat])) |
| PubMed | #1 | built environment* OR urban environment OR environment design OR urban design OR neighborhood* OR pedestrian environment OR street OR physical environment OR walkability OR residential environment OR community environment |
|  | #2 | walking activity OR physical activity OR mobility OR walking behavior |
|  | #3 | older adult* OR elderly OR seniors OR aged OR older people OR older persons OR older citizens OR late life |
|  | #4 | #1 AND #2 AND #3 |
|  | Full search string | ((built environment* OR urban environment OR environment design OR urban design OR neighborhood* OR pedestrian environment OR street OR physical environment OR walkability OR residential environment OR community environment) AND (walking activity OR physical activity OR mobility OR walking behavior)) AND (older adult* OR elderly OR seniors OR aged OR older people OR older persons OR older citizens OR late life) AND ((classicalarticle[Filter] OR comparativestudy[Filter] OR correctedandrepublishedarticle[Filter] OR evaluationstudy[Filter] OR multicenterstudy[Filter]) AND (humans[Filter]) AND (english[Filter]) AND (1970:2024[pdat])) |
| Web of Science | #1 | TS=("built environment" OR "urban environment" OR "environment design" OR "urban design" OR "neighborhood*" OR "pedestrian environment" OR "street" OR "physical environment" OR "walkability" OR "residential environment" OR "community environment") |
|  | #2 | TS=("walking activity" OR "physical activity" OR "mobility" OR "walking behavior") |
|  | #3 | TS=("older adult*" OR "elderly" OR "seniors" OR "aged" OR "older people" OR "older persons" OR "older citizens" OR "late life") |
|  | #4 | #1 AND #2 AND #3 |
| Scopus | #1 | TITLE-ABS-KEY("built environment" OR "urban environment" OR "environment design" OR "urban design" OR "neighborhood*" OR "pedestrian environment" OR "street" OR "physical environment" OR "walkability" OR "residential environment" OR "community environment") |
|  | #2 | TITLE-ABS-KEY("walking activity" OR "physical activity" OR "mobility" OR "walking behavior") |
|  | #3 | TITLE-ABS-KEY("older adult*" OR "elderly" OR "seniors" OR "aged" OR "older people" OR "older persons" OR "older citizens" OR "late life") |
|  | #4 | #1 AND #2 AND #3 |
|  | Full search string | ( TITLE-ABS-KEY ( "built environment" OR "urban environment" OR "environment design" OR "urban design" OR "neighborhood*" OR "pedestrian environment" OR "street" OR "physical environment" OR "walkability" OR "residential environment" OR "community environment" ) AND TITLE-ABS-KEY ( "walking activity" OR "physical activity" OR "mobility" OR "walking behavior" ) AND TITLE-ABS-KEY ( "older adult*" OR "elderly" OR "seniors" OR "aged" OR "older people" OR "older persons" OR "older citizens" OR "late life" ) ) AND PUBYEAR > 1966 AND PUBYEAR < 2025 AND ( LIMIT-TO ( DOCTYPE , "ar" ) ) AND ( LIMIT-TO ( EXACTKEYWORD , "Human" ) ) AND ( LIMIT-TO ( LANGUAGE , "English" ) ) |
| Embase | #1 | ('built environment' OR 'urban environment' OR 'environment design' OR 'urban design' OR 'neighborhood' OR 'pedestrian environment' OR 'street' OR 'physical environment' OR 'walkability' OR 'residential environment' OR 'community environment') |
|  | #2 | ('walking activity' OR 'physical activity' OR 'mobility' OR 'walking behavior') |
|  | #3 | ('older adults' OR 'elderly' OR 'seniors' OR 'aged' OR 'older people' OR 'older persons' OR 'older citizens' OR 'late life') |
|  | #4 | #1 AND #2 AND #3 |
|  | Full search string | ('built environment'/exp OR 'built environment' OR 'urban environment'/exp OR 'urban environment' OR 'environment design'/exp OR 'environment design' OR 'urban design' OR 'neighborhood'/exp OR 'neighborhood' OR 'pedestrian environment' OR 'street'/exp OR 'street' OR 'physical environment'/exp OR 'physical environment' OR 'walkability'/exp OR 'walkability' OR 'residential environment' OR 'community environment') AND ('walking activity' OR 'physical activity' OR 'mobility' OR 'walking behavior') AND ('older adults' OR 'elderly' OR 'seniors' OR 'aged' OR 'older people' OR 'older persons' OR 'older citizens' OR 'late life') AND [<1966-2024]/py AND [embase]/lim AND 'article'/it AND [english]/lim) |
