# Supplementary material for: Systematic review of nonlinear associations between the built environment and walking in older adults
Source: BMC Public Health. 2025 Dec 11;25:4240. doi: 10.1186/s12889-025-25141-6 (PMC12696931; doi:10.1186/s12889-025-25141-6)
Supplement: Supplementary file 3 — Supplementary Material 3. [file 12889_2025_25141_MOESM3_ESM.docx]

**Supplementary Table S3.** Scores of built environment factors

| **Dimensions** | | **Factor** | **Positive score (+)** | **Negative score (−)** | **Nonlinear score** | **Total** |
| --- | --- | --- | --- | --- | --- | --- |
| Physical factors | Density | Population density | 57 | 24 | 32 | 113 |
|  |  | Residential density | 31 | 16 | 8 | 55 |
|  | Diversity | Land-use mix | 81 | 16 | 40 | 137 |
|  | Design | Intersection density | 48 | 41 | 16 | 105 |
|  |  | Green view index | 32 | 0 | 16 | 48 |
|  |  | Sidewalk density | 24 | 0 | 24 | 48 |
|  |  | Slope | 0 | 0 | 26 | 26 |
|  |  | Normalized Difference Vegetation Index (NDVI) | 8 | 8 | 8 | 24 |
|  |  | Proportion of green space | 8 | 0 | 8 | 16 |
|  |  | Green space area | 8 | 0 | 8 | 16 |
|  |  | Pedestrian crossing facilities | 16 | 0 | 0 | 16 |
|  |  | Street density | 9 | 0 | 0 | 9 |
|  |  | Walking path length | 9 | 0 | 0 | 9 |
|  |  | Sidewalk coverage ratio | 9 | 0 | 0 | 9 |
|  |  | Sidewalk connectivity | 9 | 0 | 0 | 9 |
|  |  | Number of bicycle lanes | 9 | 0 | 0 | 9 |
|  |  | Road network density | 0 | 0 | 8 | 8 |
|  |  | Link–Node Ratio | 0 | 8 | 0 | 8 |
|  |  | Building façade details | 8 | 0 | 0 | 8 |
|  |  | Forest coverage rate | 8 | 0 | 0 | 8 |
|  |  | Green coverage ratio | 0 | 0 | 8 | 8 |
|  |  | Sidewalk quality | 8 | 0 | 0 | 8 |
|  | Destination Accessibility | Distance to parks, green spaces, and plazas | 8 | 8 | 23 | 39 |
|  |  | Number of retail facilities | 28 | 0 | 0 | 28 |
|  |  | Number of educational facilities | 8 | 17 | 0 | 25 |
|  |  | Number of parks, green spaces, and plazas | 25 | 0 | 0 | 25 |
|  |  | fitness facilities | 24 | 0 | 0 | 24 |
|  |  | Number of medical facilities | 16 | 7 | 0 | 23 |
|  |  | Density of parks, green spaces, and plazas | 17 | 0 | 0 | 17 |
|  |  | Accessibility to parks, green spaces, and plazas | 16 | 0 | 0 | 16 |
|  |  | Density of recreational facilities | 8 | 0 | 8 | 16 |
|  |  | Accessibility to public service facilities | 16 | 0 | 0 | 16 |
|  |  | Number of food service facilities | 16 | 0 | 0 | 16 |
|  |  | Accessibility to commercial facilities | 16 | 0 | 0 | 16 |
|  |  | Distance to the nearest chess & card room | 16 | 0 | 0 | 16 |
|  |  | Number of recreational facilities | 8 | 0 | 0 | 8 |
|  |  | Accessibility to retail facilities | 8 | 0 | 0 | 8 |
|  |  | Distance to the nearest commercial center | 0 | 8 | 0 | 8 |
|  | Distance to Transit | Bus stop density | 40 | 8 | 16 | 64 |
|  |  | Number of bus stops | 8 | 8 | 8 | 24 |
|  |  | Accessibility to bus stops | 8 | 7 | 8 | 23 |
|  |  | Distance to the nearest bus stop | 8 | 0 | 8 | 16 |
|  |  | Number of bike-sharing stations | 8 | 0 | 8 | 16 |
|  |  | Bus line density | 8 | 0 | 0 | 8 |
|  |  | Distance to the nearest railway station | 0 | 8 | 0 | 8 |
|  |  | Distance to the nearest subway | 8 | 0 | 0 | 8 |
| Perceived factors | | Safety | 63 | 8 | 0 | 71 |
|  |  | Aesthetics | 47 | 0 | 0 | 47 |
|  |  | Nighttime lighting | 15 | 0 | 0 | 15 |
|  |  | Natural landscape comfort | 8 | 0 | 0 | 8 |
